# Climate, Health, and Urban Green Infrastructure: The Evidence Base and Implications for Urban Policy and Spatial Planning

**DOI:** 10.3390/ijerph22121842

**Published:** 2025-12-09

**Authors:** Yirong Jia, Catalina Turcu

**Affiliations:** Bartlett School of Planning, University College London, 14 Upper Woburn Place, Central House, London WC1H 0NN, UK; yirong.jia.21@ucl.ac.uk

**Keywords:** urban green infrastructure, climate adaptation, health co-benefits, policy implication, urban planning

## Abstract

Urban green infrastructure (UGI) is widely used to adapt to the impacts of climate change. Its multiple benefits are well documented, with health-related benefits receiving growing attention, especially post-COVID-19. However, the existing evidence remains fragmented and limited to narrow disciplinary perspectives, offering only partial insights into the intersection of UGI and climate adaptation measures with health co-benefits. This paper addresses these gaps by providing an interdisciplinary review of the field. It presents a systematic literature review of studies between 2015 and 2025, assessing the extent of documented evidence and drawing out key policy implications. The review adopts the PRISMA framework and synthesizes evidence from 178 primary research articles across seven databases. Health co-benefits are reported across ten types of UGI: residential greenery, urban vegetation, school greenery, trees, urban parks, urban forests, green roofs and walls, green streets, grasslands, and community or private gardens. Building on the review’s findings and additional literature, the paper discusses seven key implications for urban policy and spatial planning, which are relevant to climate adaptation policymakers, urban planners, and public health authorities working in cities.

## 1. Introduction

Urban greening plays a key role in climate adaptation and resilience by reducing urban heat, managing stormwater, improving air quality, and supporting public health. This is reflected in the growing literature on green infrastructure, ecosystem services, and nature-based solutions—terms often used interchangeably [1]. Among these, urban green infrastructure (UGI thereafter) has drawn particular attention due to its broad applicability, enabling interdisciplinary responses to sustainability challenges.

In the absence of a unified definition [2,3,4,5,6], this paper draws on multiple definitions and refers to UGI as a network of natural and semi-natural elements functioning individually or collectively across the spatial scales of a city, and ten UGI types are considered—residential greenery, urban vegetation, trees, parks, grasslands, and so on—as defined by how UGI is measured or evaluated in the literature. UGI is commonly framed through the ‘multifunctionality thesis’, which positions it as a measure delivering multiple benefits—ecological, social, and economic—in urban settings [7,8,9]. UGI is well-recognized as a climate adaptation strategy that mitigates the urban heat island (UHI) effect by lowering air and surface temperatures through shading and evapotranspiration [10,11]; reduces runoff via interception, infiltration, retention, and rainwater storage [12,13]; captures airborne pollutants by trapping them on leaf surfaces [14]; and acts as a pollution barrier for residential areas near roads and industrial zones [15]. UGI is also recognized for its health benefits, especially post-COVID-19 [16]. It improves health by reducing exposure to air pollution, noise, and heat; enhancing biodiversity; lowering stress; and promoting physical activity [17]. Green spaces help prevent chronic diseases (e.g., diabetes, cardiovascular conditions), reduce mortality, improve pregnancy outcomes, and lower obesity [18,19,20]. They also support mental health through better cognitive function [21] and enhance well-being by increasing life satisfaction and happiness [22]. Emerging research shows that well-designed urban climate adaptation strategies can reduce health exposure and impacts, delivering co-benefits [23,24,25,26]. These health gains are often more immediate and visible than climate outcomes, strengthening the case for UGI adoption [8] and integrated and multi-benefit urban decision-making [26].

However, despite growing evidence on the concomitant climate and health benefits of UGI implementation, significant gaps remain in understanding the UGI–climate–health nexus and translating it into actionable policymaking. Evidence is often siloed, focusing on either environmental or health outcomes without addressing interactions, trade-offs, or contextual factors [26]. Closing these gaps is critical for urban governance and policymaking, where climate policymakers, urban planners, and public health authorities need to coordinate interventions and are faced with the challenge of navigating fragmented evidence to prioritize actions that deliver multiple outcomes. To address that challenge, this paper asks the following:

- *What evidence exists on the health co-benefits of UGI-based adaptation?* 
- *What are the policy implications of such evidence, especially urban policy and spatial planning?* 

The two questions are answered by a systematic literature review, which aims to consolidate evidence across urban, climate, and health studies and implications for urban policymaking—such a review was previously absent. Only two relevant systematic reviews exist. Sharifi et al. [26] examine the health co-benefits of climate adaptation via nature-based solutions but offer limited analysis of typological variation. Choi et al. [8] highlight the multiple benefits of green infrastructure and identify types (e.g., trees, shrubs, green roofs), yet health evidence is discussed in only 7 of 141 studies.

Following the introduction, Section 2 details the research design, methods, and data analysis. Section 3 reports research trends and findings from 178 studies across ten UGI types. Section 4 reviews policy evidence and supplements it with additional literature to explore implications for urban policy and spatial planning. Section 5 synthesizes key findings, highlights contributions, and identifies directions for future research.

## 2. Materials and Methods

This systematic review followed the Preferred Reporting in Systematic Reviews and Meta-Analyses (PRISMA) guidelines and was registered with the *International Prospective Register of Systematic Reviews (PROSPERO)* under registration number CRD420251173742.

### 2.1. Literature Search Strategy

This paper used the PRISMA framework, a widely recognized method for transparent and robust systematic reviews. PRISMA was selected for its adaptability and credibility, whereas alternatives (e.g., Cochrane, Campbell) are tailored to clinical or intervention trials and less suited to mixed-methods or urban environment research [27].

The search string combines UGI, climate adaptation, and health search terms, using Boolean operators (“OR” and “AND”) to ensure a comprehensive coverage. It was developed from literature reviews [8,26] and the researchers’ expertise, as follows:

- UGI terms include synonyms (e.g., nature-based solutions), types (e.g., parks, green roofs), and wildcard terms (e.g., urban green*).
- Climate adaptation terms include cognate concepts (e.g., climate change, climate crisis), UGI’s main climate benefits (heat reduction, flood management, air quality), and extreme weather events (e.g., wildfires).
- Health terms include related terms (e.g., health effects, human health), physical health, mental health, well-being, and related outcomes (e.g., diseases, mortality, depression, happiness).

The search string was refined in three steps. First, database terminology tools (e.g., MeSH in PubMed) identify relevant synonyms and variants commonly used in databases. For instance, entering “obesity” into the search box generates related terms such as “BMI.” Second, titles, abstracts, and keywords from test searches were reviewed to add further terms, using a snowball approach until no new terms emerged. For example, reviewing abstracts focused on “green wall” research revealed alternative terms “green façades” and “vertical farming.” Third, the final search string was reviewed and adjusted based on feedback from two academics and one librarian. A summarized search string is presented below and in detail in Supplementary Material—Detailed Search String.

(title OR abstract OR keywords) CONTAINS (UGI_terms) AND (Climate_terms) AND (Health_terms)

To ensure a comprehensive coverage of research on UGI, climate adaptation, and health, a total of seven databases were utilized. These included integrated databases (Web of Science, Scopus), databases focusing on spatial environment impacts on the environment (e.g., GreenFILE, GEOBASE), and databases specializing in health (PubMed, CINAHL, PsycINFO). The primary inclusion criteria restricted the search to peer-reviewed articles published in English between April 2015 and April 2025. The final literature search was conducted in June 2025. The search yielded a total of 12,087 articles: 3486 from Web of Science, 5305 from Scopus, 585 from GreenFILE, 1515 from GEOBASE, 1058 from PubMed, 85 from CINAHL, and 53 from PsycINFO.

### 2.2. Literature Screening and Selection

All references were imported into Rayyan, a web-based application designed to streamline the systematic review process by enabling efficient management, screening, and selection of research papers. Using this software, 5209 duplicate records were identified and removed, resulting in a total of 6878 documents for further manual screening. One researcher was primarily responsible for the manual screening and selection process, while another researcher independently checked and assessed the results.

The paper screening process was conducted in two rounds. In the first round, the titles and abstracts of the 6878 articles were assessed to determine their relevance to the research topic and questions.

Studies were included if they met the following criteria:

- Published in a peer-reviewed journal.
- Contained empirical data analysis (observational, experimental, or model-based).
- Examined urban green elements as distinct or independent objects, rather than as part of a composite spatial intervention.
- Included at least one climate element as a factor of investigation.
- Reported at least one health-related outcome.
- Conducted in urban contexts.

Studies were excluded if they covered the following:

- Assessed the health impacts of green elements and climatic factors separately, without exploring the interactions between them.
- Were review papers, conceptual papers, dissertations, conference proceedings, or non–peer-reviewed gray literature.

**Figure 1:**
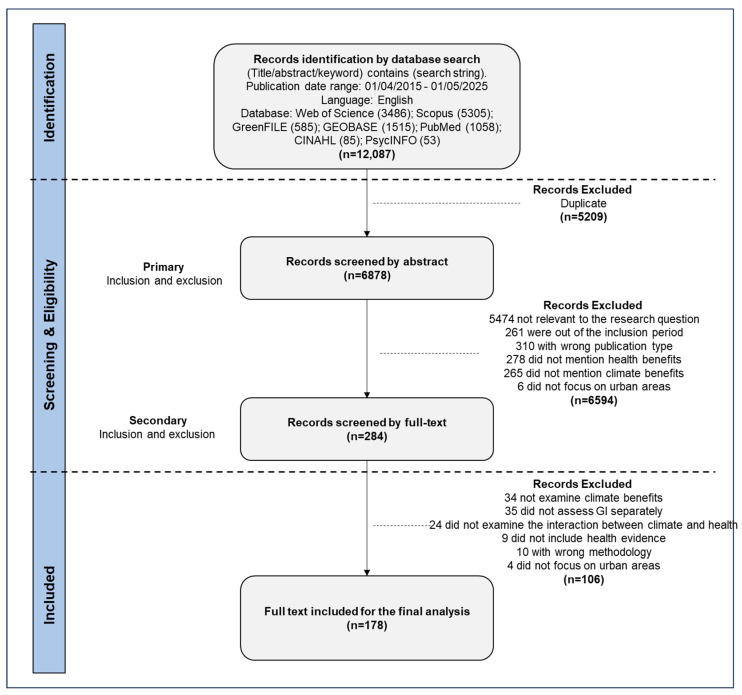
PRISMA Framework—Flowchart illustrating the literature search and selection process.

In the first round of screening, 6594 articles were excluded: 5474 for irrelevance (e.g., zoology, soil science, and microbiology articles), 261 for being out of the inclusion period, 310 for the publication type, 278 for lacking health benefits, 265 for not addressing climate adaptation, and 6 for not focusing on urban areas. In the second round, full texts of 284 papers were assessed. Of these, 106 were excluded for not meeting criteria regarding climate benefits, health evidence, UGI assessment, climate–health interaction, or urban focus. Ultimately, 178 articles were included for data extraction and analysis (see Figure 1). Included articles met the Joanna Briggs Institute (JBI) Critical Appraisal Checklist criteria for quantitative and qualitative studies [28]. Criteria assessed sampling, recruitment, group comparability, measurement validity, control of confounders, follow-up, and use of appropriate analytical methods.

### 2.3. Data Extraction and Analysis

Full texts of the remaining 178 articles were carefully read, and information about each article was collected in an Excel document, as follows:

- Publication year;
- Location of the study (city and country);
- Population group (e.g., children, adults, elderly, women, men);
- Type, scale, and measurements/indicators of UGI;
- Climate adaptation benefits covered;
- Physical health, mental health, and well-being benefits examined or resulting from the studied climate adaptation measures;
- Main research findings;
- Policy implications for spatial planning and built environment policy design.

Classifying UGI types proved challenging because interpretations differed across contexts and disciplines. Therefore, UGI types were manually synthesized through inductive content analysis, a method that is particularly appropriate and widely applied when existing knowledge on a topic is fragmented [26] and enables insights to be drawn from the literature in a comprehensive and unbiased manner, without relying on prior assumptions [29]. The initial article selected for inductive coding was the earliest study identified following the application of all inclusion criteria and the removal of duplicates. Subsequent articles were coded in chronological order. UGI elements were iteratively categorized based on descriptions in each paper about how UGI is measured, with new types created as needed. The classification process was mainly conducted by one researcher, but the decisions were cross-checked by a second researcher to ensure conceptual reliability.

### 2.4. Limitations

Although this review has made every effort to collect, select, and analyze relevant articles, it has some limitations. Some databases (e.g., Environment Complete) were excluded due to subscription restrictions, and only English-language papers were included, possibly omitting relevant studies in other languages. Nonetheless, efforts were made to minimize these limitations by incorporating a comprehensive range of search terms, informed through consultations with academics and librarians. Finally, it is noted that search strings cannot be entirely comprehensive, thus relevant articles may have been missed.

## 3. Results

### 3.1. The Longitudinal and Geographical Distribution of Studies

**Figure 2:**
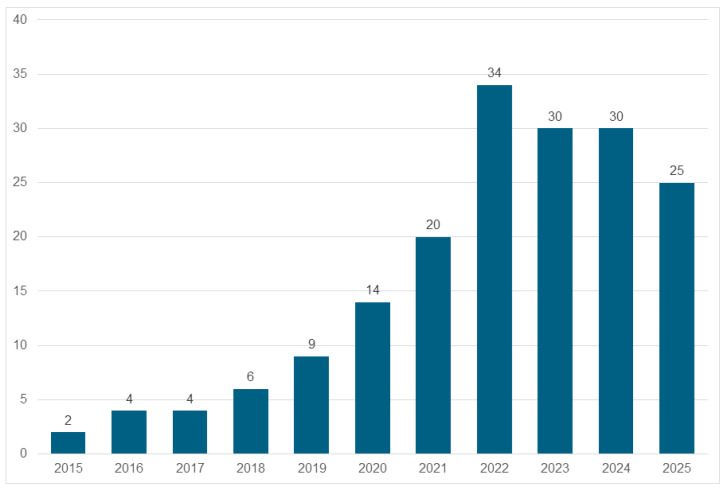
Distribution of selected publications (178) between Jan 2015 and April 2025.

Between 2015 and 2025, there has been a clear upward trend in publications focusing on UGI, climate adaptation, and health, with a peak observed in 2022 (Figure 2). One possible explanation is that the COVID-19 pandemic has stimulated public health concerns [16] and also more attention to UGI. The 2021–2025 period shows particularly high levels of research activity, possibly driven by growing interest in the intersection between urban planning, public health, and environmental science—studies collected for 2025 were published between January and April only, hence the drop that can be noted in Figure 2.

**Figure 3:**
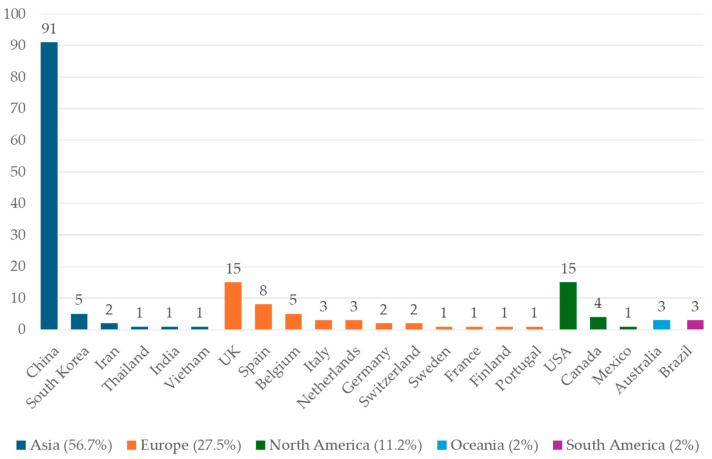
Distribution of selected studies (178) by country and continent of origin.

The studies’ geographical distribution reveals significant regional imbalances (Figure 3). Of the 178 studies reviewed, most were conducted in Asia, followed by Europe and North America—with 101, 49 and 20 studies, respectively. No studies were reported for Africa. At the country level, China contributed 91 studies—a significant outlier when compared to other countries—while the USA and UK produced 15 each. Overall, studies have focused on developed countries, with China as the notable exception. This uneven distribution frames the global relevance of findings, particularly for African and South American contexts, where rapid urbanization intensifies climate and public health challenges.

Research on China has intensified, with 94.5% of 91 articles published since 2020, largely driven by national policies promoting ecological civilization and green development under the 13th and 14th Five-Year Plans [30,31]. This surge also reflects the prolific output of Chinese scholars working in publication-oriented environments. Most studies focus on green indices near residences and schools and their associations with air pollution, heat, and chronic disease, potentially introducing a “China bias” due to limited attention to other UGI types.

Studies in the US account for 8% of this review. Most US research addresses flood management [8], but these studies lack empirical health evidence [32] and were excluded. Included studies focus on the tree canopy, green roofs, and street trees, examining links between UGI, air pollution, urban heat islands, extreme temperatures, and health outcomes such as cardiovascular disease, heat-related illness, and mental health. The UK leads European UGI research, while fewer studies from France, Germany, and Italy may reflect language barriers. European research employs diverse methods, increasingly evaluates green quality, explores additional pathway mechanisms, and emphasizes vulnerable groups and mental health.

### 3.2. Classification of Studies by UGI Type and Scale

Given the varied interpretations of UGI shaped by national contexts, research objectives, and disciplinary perspectives, this review classifies UGI types and scales based on the methodological approaches used for their measurement and evaluation (Table 1). These categories were derived through inductive content analysis. 

**Figure 4:**
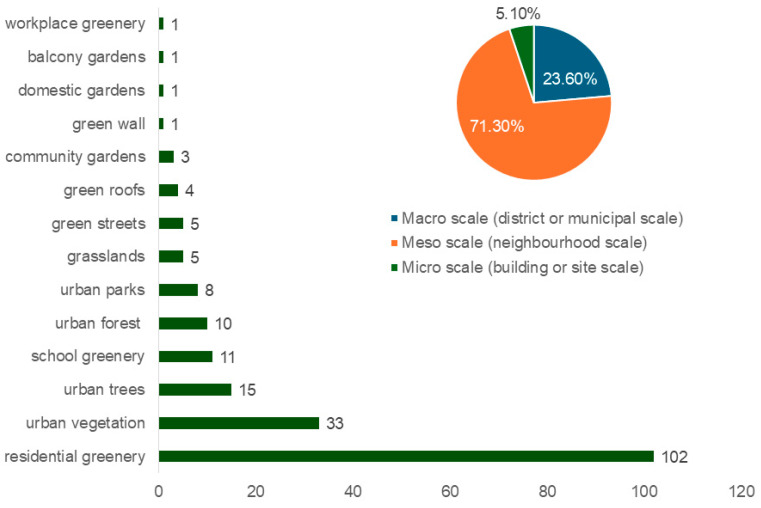
Distribution of selected studies (178) by UGI type and spatial scale.

Overall, the top three UGI types examined are residential greenery, urban vegetation, and urban trees—with 102, 33, and 15 studies, respectively (Figure 4). Other types, ranked by a decreasing number of papers, include school greenery, urban forests, urban parks, grasslands, green streets, green roofs, and community gardens. 

This study defines the UGI scale as macro, meso, or micro following Barker et al. [33]. Most studies examined the meso scale (71.3%), followed by the macro (23.6%) and micro (5.1%) scales (Figure 4). This is most likely a reflection of policy and evaluation focuses on interventions at the macro and meso scales, as their climate and health benefits are easiest to measure [34].

**Figure 5:**
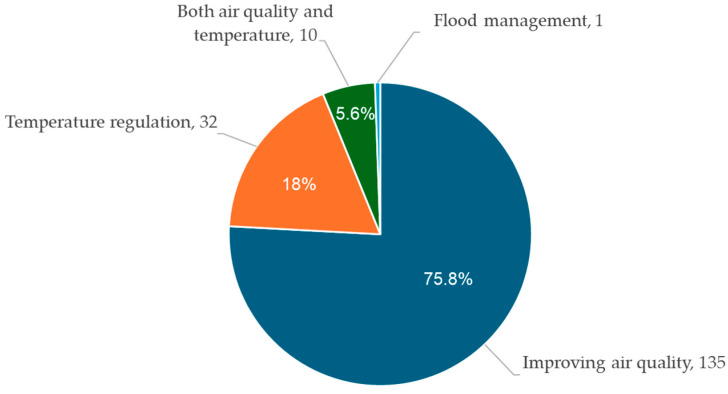
Distribution of selected studies by type of climate adaptation pathway.

Most studies on UGI climate adaptation pathways addressed air quality (75.8%) or heat reduction (18%), with few examining both and one examining flood management (Figure 5).

**Figure 6:**
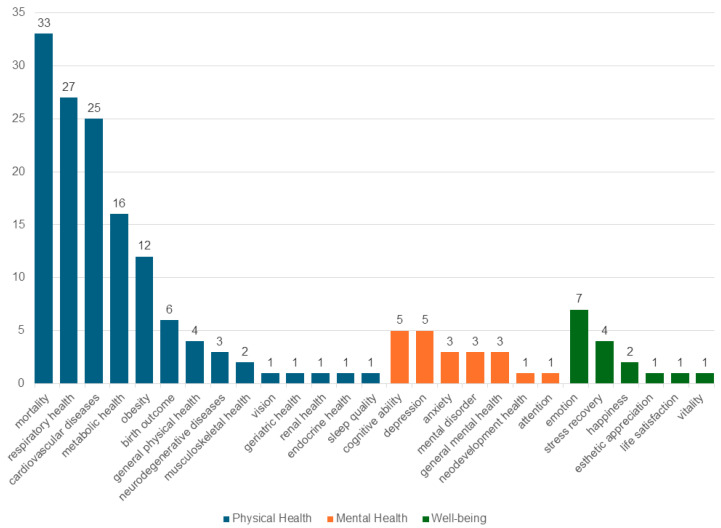
Distribution of selected studies by type of health co-benefits.

Regarding health co-benefits, 82% focused on physical health (mainly mortality, CVDs, respiratory and metabolic health), followed by mental health (11.2%) and well-being (8.4%) (Figure 6).

### 3.3. The Evidence of Climate Adaptation with Health Co-Benefits Across UGI Types

Ten UGI types—as defined in Table 1—are discussed in this section in order of their ‘popularity’ in research, as shown in Figure 4.

**Figure 7:**
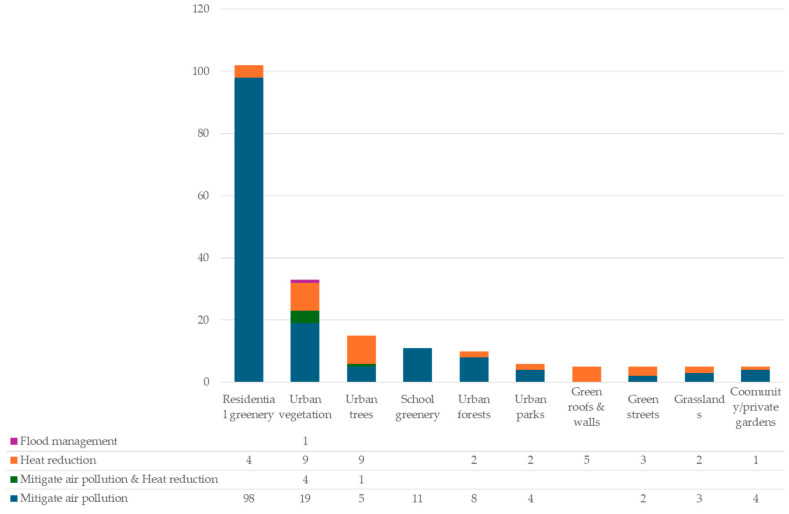
Ten UGI types by climate adaptation pathways with health co-benefits.

Figure 7 shows their distribution across climate adaptation pathways associated with health co-benefits. Across all UGI types, the most documented pathways are air pollution (135 studies) and heat mitigation (32 studies), or a combination of both (10 studies), with only one study looking at flood management.

**Figure 8:**
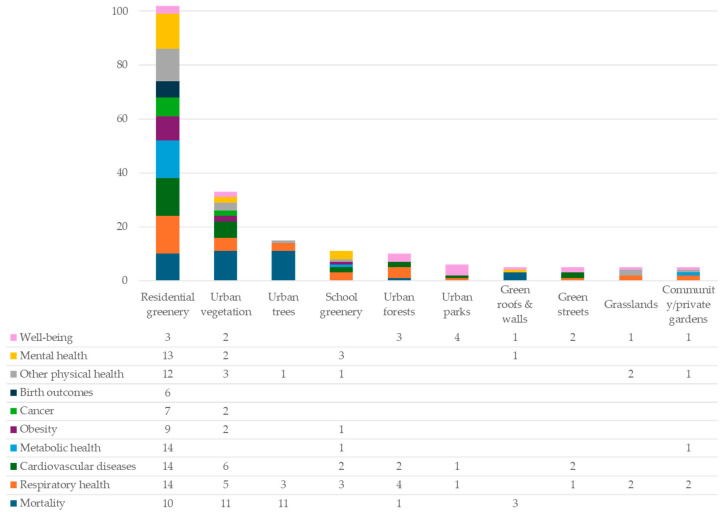
Ten UGI types and associated health co-benefits.

Figure 8 indicates what types of health co-benefits they are associated with—see the Supplementary Tables S1 for further detail. Physical health benefits are overwhelmingly documented across all UGI types (146 studies), followed by mental health (19 studies) and well-being (17 studies).

#### 3.3.1. Residential Greenery

Residential greenery is the most studied UGI type (102 studies, 57%), typically measured as neighborhood-level greenness using NDVI or green space coverage within 150–1000 m of residences. Most studies treated residential greenery as a meso-scale exposure, encompassing trees, parks, gardens, and green roofs. Residential greenery is mainly linked to physical health outcomes (86 studies)—such as mortality (ten studies), cardiovascular diseases (fourteen studies), respiratory health (fourteen studies), metabolic health (fourteen studies), obesity (nine studies), cancer (seven studies), birth outcomes (six studies), other physical health (twelve studies), mental health (thirteen studies), and well-being (three studies).

*Mortality*—Ten studies found that residential greenery reduces mortality by lowering air pollution and heat exposure. Nine reported decreased non-accidental and cause-specific mortality, particularly among youth and women, by reducing PM_2.5_, PM_10_, and NO_2_ exposure [35,36,37,38,39,40,41,42,43]. One study showed that greater greenery (NDVI within 250 m) lessened heat-related mortality among those 65 and older [44], especially in warmer southern Chinese cities, emphasizing the importance of the local climate and health context in planning.

*Cardiovascular diseases (CVDs)*—Residential greenery reduces the cardiovascular risk by improving the air quality and lowering thermal stress. Reduced exposure to particulate matter and traffic pollution lowers risks of stroke [45,46], hypertension [47,48,49,50,51], and progression of coronary [52,53,54,55] and ischemic heart disease [56], with effects stronger among women and adults under 65 [46,53]. It also buffers temperature extremes, lessening cardiovascular strain during heatwaves [55,57] and cold spells [58]. Larger, connected, and aggregated green patches are most effective [55].

*Respiratory health*—Residential greenery affects respiratory health through two contrasting pathways: improving air quality and lowering PM_2.5_, NO_2_, and O_3_, which are linked to reduced respiratory mortality [59], lower asthma prevalence [60,61,62,63], fewer hospital admissions [64,65], better lung function [66,67], and higher tuberculosis treatment success [68]. Benefits are strongest when residential greenery is dense or clustered, which enhance pollutant deposition and airflow buffering [62]. However, some studies link it to an increased risk of asthma [69,70], respiratory symptoms [63,71], and declining lung function [72], especially in children, due to allergenic pollen or vegetation trapping pollutants [69,71]. These negative effects are more common in low-diversity areas with dominant allergenic plants, with increased biodiversity noted to reduce pollen concentrations, enhance the ecological balance [61,69,70], and support immune regulation in children to lower inflammatory and allergic risks [73,74].

*Metabolic health*—Residential greenery is linked to improved metabolic health, by reducing air pollution exposure, associated with lower diabetes risks [75,76,77,78], gestational diabetes [79,80,81], metabolic syndrome [82,83,84,85], fatty liver disease, and abnormal lipid profiles [86,87,88]. These benefits result from vegetation moderating inflammation and oxidative stress caused by PM_2.5_, PM_10_, NO_2_, and O_3_. Benefits are stronger for diabetes than metabolic syndrome [82,83,84,85], and they are likely to diminish in areas with very high pollution, where greenery loses its buffering capacity [77,83].

*Obesity*—Residential greenery is associated with lower obesity rates, mainly through reduced exposure to PM_1_, PM_2.5_, PM_10_, and NO_2_, which supports better weight regulation and a lower BMI [89,90,91,92,93,94,95,96,97]. Effects are stronger among women and lower socioeconomic groups [90,92,93,94]. However, like results for metabolic health, in areas with high pollution, greenery’s protective impact diminishes or reverses, indicating a threshold where benefits occur only if vegetation sufficiently buffers local pollution [91].

*Cancer*—Residential greenery may protect against certain cancers by reducing exposure to PM_2.5_, PM_10_, and NO_2_. The strongest evidence is for lung cancer, with lower particulate levels linked to a reduced incidence and mortality [98,99,100,101]. Findings for breast cancer are mixed, with some studies showing benefits from lower pollution [98,102] and better mental health [103], but others noting weaker or context-dependent effects [101]. Reduced PM_10_ exposure is also linked to a lower risk of oral cavity, pharynx, and non-melanoma skin cancers [104]. These results highlight the importance of addressing air pollutants, particularly PM_10_, in cancer prevention.

*Birth outcomes*—Residential greenery improves birth outcomes by reducing maternal exposure to air pollution and heat. Lower PM_2.5_, PM_10_, NO_2_, and SO_2_ levels are linked to fewer preterm births, higher birth weights [105,106,107,108], and a lower atopic dermatitis risk [109]. Residential greenery also protects against heat-related miscarriage risk [110]. Benefits are greatest in urban areas with high pollution or heat, where it can significantly reduce environmental exposures.

*Other physical health*—Emerging evidence reports associations with improved general health [111,112], lower risks of neurodegenerative diseases [113,114,115], better musculoskeletal [116,117] and visual health [118], reduced frailty [119], lower risks of kidney [120] and liver disease [121], and fewer thyroid nodules [122]. This emerging evidence suggests that it may reduce systemic inflammation and oxidative stress by mitigating exposure to PM_2.5_, PM_10_, NO_2_, and O_3_.

*Mental health and well-being*—Residential greenery supports mental health by reducing exposure to traffic-related pollutants (PM_2.5_, NO_2_), in turn lowering risks of neurodevelopmental disorders in children, including improved attention [123,124], working memory [21], and cognitive development [125] and a lower autism risk [126]. In adults, it is linked to reduced depression and anxiety rates [127,128,129,130,131,132,133,134], especially in moderately polluted areas. It also enhances subjective well-being, life satisfaction, and stress recovery by improving perceived air quality and social cohesion [135,136,137].

#### 3.3.2. Urban Vegetation

Urban vegetation is the second most studied UGI type (33 studies, 19%), typically assessed as overall greenness using the NDVI or green coverage, irrespective of the vegetation type. Most studies focused on macro-scale UGI interventions and investigated health outcomes including mortality (eleven studies), cardiovascular disease (six studies), respiratory health (five studies), obesity (two studies), cancer (two studies), other physical health (three studies), mental health (two studies), and well-being (two studies).

*Mortality*—Urban vegetation is linked to lower mortality by mitigating urban heat island effects [138,139,140,141,142,143,144,145,146]. An increased tree canopy and vegetated areas can reduce heat exposure at city scales [143,144]. Clustered vegetation further enhances the cooling effect [145] and particularly benefits older adults [141,142] and those in heat-vulnerable neighborhoods [143]. Some studies also associate urban vegetation with lower mortality via reduced exposure to PM_2.5_ and PM_10_ [139,147,148], and with stronger air quality benefits in lower socioeconomic areas [147].

*Cardiovascular diseases (CVDs)*—Urban vegetation benefits cardiovascular health by reducing exposure to PM_2.5_ and PM_10_, which is achieved by lowering the heat pathway. These effects are linked to lower hypertension [149,150], fewer CVD hospital admissions [151], reduced CVD mortality [152,153], and less heat stroke [154]. Benefits vary by age: adults 65–84 benefit most from a better air quality [151], while those ≥85, especially women, are more sensitive to cooling [150]. Larger, connected green patches offer greater cardiovascular protection than fragmented greenery [153], highlighting the importance of the spatial configuration.

*Respiratory health*—Urban vegetation can both protect and harm respiratory health. Greater vegetation cover lowers PM_2.5_ and PM_10_, reducing respiratory-related hospital admissions [151], mortality [155], and sometimes infection transmission [156]. However, increased pollen exposure or poor air circulation can raise childhood asthma [157] and allergic rhinitis risks [158]. Larger, cohesive vegetation patches improve air quality, while fragmented or poorly selected greenery may heighten allergenic or pollution-related risks [155].

*Obesity*—Evidence for urban vegetation reducing obesity is limited but suggests that lower PM_2.5_ and PM_10_ levels may decrease obesity by alleviating systemic inflammation and related metabolic burdens [159,160]. However, these benefits are highly sensitive to baseline pollution levels: when overall pollution is very high [159] or when multiple pollutants co-occur [160], the protective effect of urban vegetation is substantially weakened.

*Cancer*—Studies examining cancer outcomes similarly indicate positive associations driven by decreased exposure to particulate pollutants, with reduced risks observed for lung [161] and breast cancers [162]. Some evidence suggests that these benefits can exhibit spatial spillover effects, where vegetation in adjacent or surrounding areas contributes to risk reduction [162]. This highlights the importance of green network connectivity and cross-boundary ecological continuity, rather than focusing solely on vegetation within administrative or neighborhood boundaries.

*General physical health*—A smaller number of studies link urban vegetation to improvements in general physical health, mainly through reducing ambient air pollution. Lower levels of PM_2.5_, PM_10_, SO_2_, and O_3_ are associated with better general regional residents’ health [163] and self-reported physical health [164] and with less oxidative stress [165]. However, these effects vary across cities. Benefits are clearer in regions with moderate baseline pollution. In areas with consistently high pollution, vegetation often does not reduce exposure enough to make a difference [163]. This shows that, at the urban scale, the health effects of vegetation depend on whether air quality improves enough to change background conditions.

*Mental health and well-being*—Evidence for mental health and well-being benefits from urban vegetation is limited and inconsistent. Some studies report reduced anxiety [166] and improved cognitive function among older adults [167], mostly due to lower heat exposure and reduced particulate pollution. Other research links urban vegetation to greater life satisfaction and happiness, particularly where it improves perceived air quality [168] or reduces flooding [169]. However, these benefits depend on vegetation being cohesive and accessible, as fragmented green patches may increase anxiety [166] and reduce life satisfaction [168], indicating that visible greenery alone is not enough; psychological benefits arise when vegetation is experienced as coherent, accessible, and supportive of restorative or social environments.

#### 3.3.3. Urban Trees

This group of fifteen studies (8%) accounts for the third most documented area of research. These studies isolate urban trees—tree coverage, canopy coverage, density, and quantity—to specifically assess their climate adaptation and health impacts across the meso- or macro-scale. Health outcomes concentrate on mortality and morbidity (eleven studies), respiratory health (three studies), and sleep quality (one study).

*Mortality and morbidity*—Urban trees are strongly associated with reduced heat-related mortality and morbidity, primarily through their cooling effect on the urban heat island (UHI) [143,170,171,172,173,174,175,176,177]. Increasing the tree canopy cover has been shown to lower city temperatures and substantially reduce heat-related deaths, with the largest benefits observed among older adults [175] and in cities with a low baseline tree coverage and significant UHI effects [174]. For instance, in London, Taylor et al. estimated that between 2015 and 2022, urban trees prevented 16% of total heat-related mortality. Increasing the tree coverage by 10% could reduce UHI-related mortality by another 10%, and maximizing the tree coverage could reduce this mortality by 55% [172]. Compared with other types of UGI, urban trees provide the most effective cooling performance [143,170], reflecting their shading capacity and evapotranspiration efficiency. A smaller number of studies also report reductions in non-accidental and cause-specific mortality through the mitigation of particulate and traffic-related air pollution (PM_2.5_, PM_10_, NO_2_, O_3_) [177,178,179]. 

*Respiratory health*—Urban trees provide consistent protection for respiratory health, more so than several other UGI types. By reducing PM_2.5_, NO_2_, O_3_, and SO_2_ concentrations, a higher tree canopy density is linked with fewer asthma hospitalizations and respiratory symptoms [60,62,180]. This benefit persists even when baseline pollution is high [60]. In contrast, the advantages of larger parks or general green spaces become inconsistent under severe pollution [60]. Additionally, dense or clustered tree patches are especially effective [62]. This suggests that the vegetation structure and canopy volume are more important than the total green area alone for respiratory outcomes. 

#### 3.3.4. School Greenery

Eleven studies (6% of the total) included in this review examined the health influence of a school with surrounding greenness on children and adolescents, defined as the assessment of overall greenness (NDVI or green space coverage) within a buffer area around schools. The associated health evidence is focused on cardiovascular diseases (two studies), respiratory health (three studies), metabolic health (one study), obesity (one study), vision (one study), cognitive abilities (two studies), and general mental health (one study).

Evidence on school greenery consistently points to health benefits for children, which occur mainly by reducing exposure to traffic-related air pollution. Reduced concentrations of PM_2.5_, PM_10_, PM_1_, and NO_2_ are linked to lower risks of hypertension [47,181], respiratory symptoms [63,71], and asthma [182]. Improved metabolic [183], obesity [184], and visual health [118] outcomes have also been observed. Benefits are particularly strong among children from lower-income families [47] or with lower levels of parental education [183]. School greenery is further associated with better cognitive development [21,185] and improved well-being [186], benefits that reflect both reduced exposure to pollutants and increased opportunities for psychological restoration during daily school activities.

#### 3.3.5. Urban Forests

Ten studies (6% of the total) in this review focus on urban forests. They either focus on specific urban forests or isolate urban forests within urban green spaces, and these are typically examined at the meso- (neighborhood scale) and macro-scales (district scale). The evaluation indicators involved are more diverse, including urban forest proximity, presence of urban forests, forest greenness, and percentage cover of forest-type green spaces. The collected health evidence surrounds respiratory health (four studies), cardiovascular diseases (two studies), mortality (one study), and well-being (three studies).

*Respiratory health*—Urban forests provide consistent respiratory health benefits, largely due to their higher volume of canopy and their ability to filter pollutants. By reducing concentrations of PM_2.5_, PM_10_, NO_2_, and O_3_, urban forests are linked to lower risks of respiratory symptoms and related morbidity [187,188,189,190]. For instance, Nowak et al. found that urban forests in 86 Canadian cities removed a total of 16,500 tons of air pollution in 2010, preventing 30 human deaths and 22,000 cases of acute respiratory symptoms, while generating an estimated USD 227.2 million in health-related benefits [187].

*Cardiovascular diseases (CVDs) and mortality*—Urban forests may offer cardiovascular and mortality benefits, though findings are more limited and often based on short-term exposure studies. Temporary reductions in cardiovascular risk factors [191] and lower CVD-related mortality during periods of high temperature [192] have been linked to reduced exposure to air pollution and environmental stressors, such as noise and heat, during forest visits. Additionally, areas with denser forest greenness have shown lower all-cause mortality, especially for heart- and lung-related conditions—by mitigating particulate pollution (PM_2.5_ and PM_10_) [193].

*Well-being*—Short-term exposure to urban forests is consistently associated with improved emotional well-being and faster stress recovery, primarily through reduced air pollution exposure and enhanced thermal comfort. By lowering concentrations of PM_2.5_ and PM_10_ [194,195] and moderating ambient temperature [196], forest environments alleviate mood disturbances, promote a positive affect, and support psychological restoration. These benefits are particularly evident under moderate pollution and temperature conditions [195], suggesting that forest visits are most restorative when environmental stressors are not extreme.

#### 3.3.6. Urban Parks

Eight studies (5% of the total) in this review examined urban parks. Similar to urban forests research, they typically focused on meso- or macro-scale climate adaptation and health impacts, with assessment indicators such as the number and size of urban parks and park accessibility. The resulting useful health evidence includes well-being (four studies), cardiovascular diseases (one study), and respiratory health (one study).

*Well-being*—Urban parks consistently promote positive emotions, facilitate stress recovery, and enhance overall well-being, primarily through air pollution mitigation [135,197] and the regulation of thermal comfort [198,199]. Tree canopies deliver the most significant cooling effects within park environments, and visitors typically prefer shaded, cooler areas that encourage relaxation [198]. These benefits are especially evident among younger individuals, whose well-being is more responsive to variations in thermal and environmental comfort [198,199]. Additionally, the park configuration influences psychological responses; semi-open or semi-enclosed layouts generally provide greater restorative benefits than fully open or closed spaces, likely because they offer a balanced sense of openness and shelter [197].

*Other health outcomes*—Beyond psychological well-being, evidence linking urban parks to physical health outcomes is limited and inconclusive. Some studies report potential respiratory [200] and cardiovascular [191] benefits due to reductions in particulate pollutants (PM_2.5_ and PM_10_); however, these effects are inconsistent and highly dependent on specific contexts. Additionally, research investigating the relationship between the park proximity and rates of obesity [91] or depression [127] has found no significant associations, suggesting that accessibility alone does not guarantee measurable climate adaptation or health improvements.

#### 3.3.7. Green Roofs and Green Walls

This review includes four articles focusing on macro-scale green roofs (e.g., green roof coverage, area, or scenario) and one study on micro-scale green walls (green wall size), with associated health outcomes including respiratory health (three studies), mental health (one study), and well-being (one study).

Although evidence on green roofs and walls remains limited, current research identifies heat mitigation and thermal regulation as the primary health pathways. Most studies associate green roofs with reduced heat-related mortality, primarily through lowering indoor temperatures during heatwaves [170,201,202]. These benefits are projected to increase under future warming scenarios [201]. The effectiveness of green roofs varies by local temperature thresholds for heatwave mortality, showing a strong regional dependence [170,201]. Integrative designs, such as green roofs combined with tree planting and other vegetated surfaces, further enhance cooling and health protection [170]. Additionally, one study identified that green roofs, specifically rooftop “urban food forest” planting beds, could reduce the risk of depression through heat reduction [203]. As for green walls, only one study was included and demonstrated their potential to boost positive emotions, reduce stress, and slightly improve cognitive abilities by regulating thermal comfort [204].

#### 3.3.8. Green Streets

Five studies in this review focused on green streets either exclusively or as part of the broader scope at the meso-scale (e.g., street view greenery, green view index, size, species, and condition of street trees), providing health evidence related to cardiovascular diseases (two studies), respiratory health (one study), and well-being (two studies).

Green streets—particularly those with a substantial tree canopy—contribute to cardiovascular and respiratory health primarily by reducing heat and mitigating air pollution. By lowering exposure to high temperatures [192] and PM_2.5_ [205], green streets are associated with reduced cardiovascular mortality and morbidity, with protective effects reported to be more pronounced among women, who appear more sensitive to environmental stressors. Street trees can also lessen respiratory risks by reducing particulate pollution, although the presence of certain allergenic species may increase asthma hospitalizations [206], underscoring the importance of species selection. Additionally, green streets enhance thermal comfort through shading, cooler ambient temperatures, higher relative humidity, and reduced wind speeds, which support psychological restoration and improved subjective well-being [207,208].

#### 3.3.9. Grasslands

Only five studies included in this review examined the meso-scale health benefits of grasslands and shrublands (e.g., grassland coverage), despite these being a relatively common form of green space in many European countries. The associated health outcomes include general physical health (two studies), respiratory health (two studies), and well-being (one study).

Evidence on grasslands shows mixed health effects. On the one hand, grasslands can support general physical health benefits (e.g., morbidity, sleep quality) by mitigating air pollution and heat reduction, while also delivering substantial economic value [178,209]. On the other hand, respiratory health findings reveal potential adverse effects, particularly higher risks of childhood asthma in areas dominated by low-diversity or monoculture lawns [180,190], suggesting that vegetation homogeneity may amplify allergenic exposure. Grasslands may also contribute to psychological restoration by improving thermal comfort, though typically less effectively than tree-covered environments [196]. In summary, the health impacts of grasslands are largely determined by their plant diversity and ecological structure, suggesting that grasslands operate as open, exposure-sensitive UGI in which both benefits and risks coexist.

#### 3.3.10. Community or Private Gardens

Evidence in gardens—both community (three studies) and private (two studies)—is limited but suggests several health-related benefits. Community gardens can reduce respiratory risks by lessening exposure to NO_2_, PM_2.5_, PM_10_, and SO_2_ [60,190], though these effects shrink when pollution is high [60]. Shaded features like trees and climbing plants help cool the area and improve comfort, supporting positive moods and emotional recovery [210]. Private gardens also help mitigate air pollution, and studies connect domestic and balcony gardens to lower risks of diabetes [77] and other pollution-linked health issues [211]. Overall, community gardens serve as small-scale, person-proximal green spaces; the associated health effects are strongly influenced by local pollution levels, vegetation structure, and patterns of daily exposure.

## 4. Policy Evidence and Implications for Urban Policy and Spatial Planning

**Figure 9:**
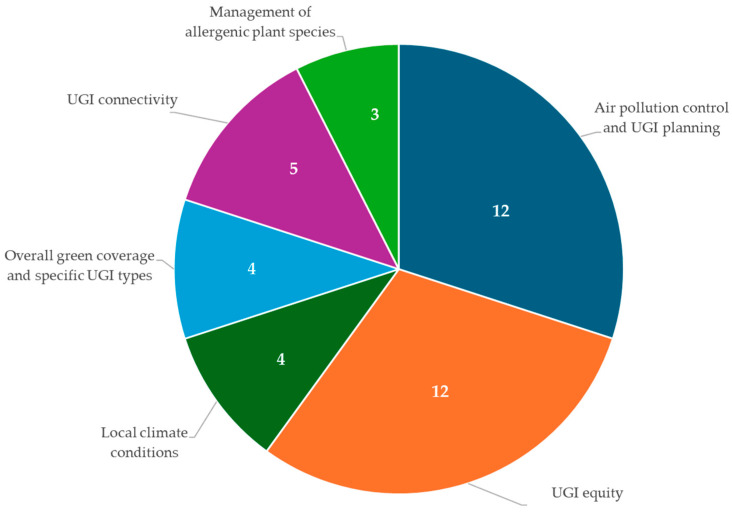
Policy areas where UGI’s climate and health benefits intersect: air pollution control and UGI planning (12 studies: [45,50,60,68,89,122,129,158,187,189,194,200]); UGI equity (12 studies: [44,101,107,120,136,138,171,174,175,198,201,209]); local climate conditions (4 studies: [44,161,163,202]); overall green coverage and specific UGI type (4 studies: [102,127,179,208]); UGI connectivity (5 studies: [68,145,153,155,212]); and management of allergenic plant species (3 studies: [69,190,206]).

Of the 178 articles reviewed, 38 (21%) presented evidence that is relevant to policymaking. Figure 9 shows the distribution of these studies by policy areas where UGI’s climate and health benefits intersect.

When examining how existing evidence supports policymaking for UGI adaptation with health co-benefits, several policy-relevant themes emerged by UGI type:

- *Residential greenery* and *urban vegetation* can reduce air-pollution-related mortality and cardiovascular disease in younger individuals and women [38,40], and they can lower heat-related mortality in older adults [44,150]; however, benefits diminish in highly polluted areas [71,91,163], depend on the scale and density of urban areas [62,155], and may exacerbate allergenic reactions in children [69,157].
- *School greenery* is linked to improved child health via air pollution mitigation, especially in deprived urban areas [118,183].
- *Urban trees* offer significant health benefits (mortality, respiratory health) by reducing air pollution and mitigating heat [60,143].
- *Urban forests* provide health benefits (cardiovascular and respiratory health, well-being) by mitigating air pollution through a high tree density [187,192].
- *Urban parks* enhance well-being by improving thermal comfort, especially in younger users [198,199], and offering restorative effects in semi-enclosed spaces [197].
- *Green roofs* and *walls* help mitigate urban heat, particularly in cities with lower heatwave mortality thresholds [170,201].
- *Green streets* promote walkability and support physical health and well-being [206,207], while *grasslands* offer a low-cost health-promoting alternative [178].

Drawing on this evidence and additional literature that highlights the role of urban policy and spatial planning in advancing climate adaptation with health co-benefits, seven key policy implications are discussed below.

**1. Smart greening: Balancing coverage with targeted typologies.** Macro-scale UGI types (e.g., residential greenery, urban vegetation) consistently improve health outcomes—such as lower mortality, reduced cardiovascular and respiratory diseases, fewer metabolic disorders, cancer, and adverse birth outcomes, alongside better mental health and well-being. Setting explicit greenness targets in urban planning is essential for tracking environmental and health benefits and return on investment [9]. However, focusing solely on overall greenness may obscure the distinct advantages of specific UGI types. Policymakers should adopt a dual approach: integrate city-wide greenness targets into strategic planning for monitoring and evaluation, and embed requirements for specific UGI types into zoning and design codes to address local climate and health priorities [213,214]. Urban planners and municipalities can lead this shift by revising spatial planning frameworks that combine overall coverage targets with type-specific interventions.

**2. Green prescribing: Tailoring UGI to health needs.** To realize its full co-benefits, climate adaptation must align with local health priorities through context-sensitive planning and stronger collaboration with public health authorities. Strategies should match interventions to health profiles—for example, prioritizing tree cover and green roofs in heat-prone areas [170]; creating high-quality parks and urban forests where depression rates are high [128,195,196]; and reducing traffic-related air pollution exposure in neighborhoods with vulnerable children while avoiding allergenic, low-diversity grasslands [182,190,206]. This requires integrating health data into spatial planning [215]. Urban planners should revise spatial frameworks to embed health-informed UGI standards, and public health agencies should provide targeted data and guidance to support these decisions.

**3. Health in the links: Prioritizing UGI connectivity.** UGI connectivity can strengthen health outcomes by reducing air pollution exposure and heat-related mortality [62,145,155]. Fragmented UGI, by contrast, increases risks of respiratory and cardiovascular diseases [153,155]. The evidence of a 20% reduction in cancer risk linked to connected *urban vegetation* [161] highlights the need for integrated networks rather than isolated patches of UGI. Urban planners and municipalities should embed UGI connectivity standards into spatial planning and zoning regulations, replacing fragmented approaches with coordinated UGI networks that deliver measurable health and climate resilience gains.

**4. No one-size-fits-all: Adapting UGI to local climates.** While UGI’s protective effects against heat-related mortality are strongest in warmer regions with pronounced UHI effects [44,174], colder climates require different strategies. Urban policy must prioritize the tree canopy, cooling spaces, and expanded parks in heat-prone areas [216] while focusing on windbreaks, evergreen species, and seasonally adaptive vegetation in colder regions. National and regional greening guidelines should mandate climate-sensitive targets for vegetation types, planting densities, and spatial strategies [217,218]. Urban planners should revise local planning frameworks to incorporate these standards, and government and public health bodies should update their guidance to ensure flexibility and enforceable climate-responsive UGI requirements.

**5. Greening pollution: Integrating UGI with air pollution management.** While UGI can reduce exposure to pollutants such as PM_2.5_, PM_10_, NO_2_, SO_2_, and O_3_, its effectiveness declines in areas with high pollution or complex pollutant mixes, reversing the benefits for respiration, metabolism, and mental health [60,159,163]. Local authorities must assess pollutant concentrations before intervention and adopt targeted planting strategies—such as rough-leaved evergreens for PM capture [219], broadleaf species for NO_2_ [220], and low-BVOC emitters for O_3_ [221]. In heavily polluted zones, UGI should be combined with emissions control and sustainable transport measures [222]. Urban planners should integrate real-time monitoring and adaptive greening into smart city systems, following models like Breathe London [223]. National regulators must update greening guidelines to require pollutant-specific planting standards and mandate coordination between urban planning and air quality management.

**6. Inclusive greening: Embedding social and health equity into UGI planning.** Health benefits vary by socioeconomic and demographic characteristics: older adults face higher heat-related and cardiovascular risks [44,141], women benefit more in terms of cardiovascular and metabolic benefits [40,205], children are vulnerable to allergenic species [70,190], and youth well-being depends on thermal comfort [198,199]. Low-income groups often experience greater metabolic health gains from UGI [39,147,183]. To address these disparities, planners can map vulnerable populations, tailor interventions to their needs, and ensure equitable access to and use of UGI [224]—for example, shaded seating for older adults and low-pollen greenery for families. Municipalities should mandate equity-based design standards in planning frameworks, and public health agencies should provide data to guide these interventions. Inclusive co-design with affected communities should become a requirement, ensuring UGI delivers climate resilience, health benefits, and social inclusion [225].

**7. Long- vs. short-term planning: Aligning UGI, climate, and health timelines.** Health benefits unfold over different timescales: heat-related outcomes such as reduced mortality and cardiovascular disease and improved well-being often emerge within one year [44], while benefits from air pollution mitigation—such as improved respiratory and metabolic health and reduced cancer risk—require five years [139,162]. Short-term planning alone risks undervaluing UGI’s long-term health returns [226]. Urban planners and municipalities should integrate adaptive strategies across timescales into planning frameworks [8]—for example, prioritizing urban forests for immediate cooling and long-term air quality improvements—and national policymakers should revise funding and evaluation criteria to recognize both short- and long-term health gains of UGI.

## 5. Conclusions

This paper has systematically reviewed the literature on the health co-benefits of climate adaptation through UGI, adopting an interdisciplinary lens and focusing on evidence-based links between UGI types, climate pathways, and associated health outcomes, of relevance to policymakers—this is summarized in Table 2.

The paper makes three key contributions. First, it advances methodological rigor through an interdisciplinary search strategy—combining snowballing, synonym expansion, and expert validation—across seven databases covering environmental, health, and spatial disciplines. This consolidation provides a robust basis for integrated UGI evidence-based climate adaptation with health co-benefits. Second, it synthesizes knowledge that is relevant to policymakers who engage in the transformation of the spatial and built environment, while advancing an underdeveloped body of interdisciplinary research at the UGI–climate–health nexus. Third, it develops further policy evidence to derive actionable implications for urban policy and spatial planning, addressing a current lack of specificity and practical guidance in existing studies.

Finally, key areas for future research are highlighted. **1/**Research on health trade-offs of UGI adaptation is limited. The review identified three areas of immediate concern: the links between allergenic species and children’s asthma risk [69,70,190,206]; UGI’s inefficacity or health risk in areas with high air pollution [157,159,160]; and negative health impact of fragmented UGI [153,155]. **2/**Evidence linking UGI, the flood management pathways, and health is scarce [32,169]. This gap is disciplinary [227], but also framed by a methodological challenge [228] and predominance of gray infrastructure approaches [229]. **3/**Most studies use NDVI to measure UGI coverage; however, NDVI overlooks the vegetation type, structure, use, and quality, factors critical for health outcomes [47,138,141]. Future research should combine multiple metrics [75] with subjective measures like the perceived green space quality [93]. 

## Figures and Tables

**Table 1:** UGI assessment indicators and associated UGI type classification.

Evaluation Measure and Indicators	UGI Type
NDVI, EVI, green coverage (within 150–1000 m buffers around residences)	Residential greenery
NDVI, green coverage (within specific urban area)	Urban vegetation
Tree canopy coverage, density, and quantity	Urban trees
NDVI, green coverage (within 150–1000 m buffers around schools)	School greenery
Forest proximity; presence of urban forests; forest coverage and greenness	Urban forest
Park accessibility; number and size of urban parks	Urban parks
Green roof coverage, area, or scenario; green wall size	Green roofs and walls
Street view greenery; green view index; size, species, and condition of street trees	Green streets
Grassland coverage	Grassland
Garden coverage; green configuration	Community gardens

**Table 2:** UGI types, climate pathways, health benefits, and contextual factors—which determine variation in outcomes.

UGI Type	Climate Pathway	Health Benefits (↓ Reduced; ↑ Increased)	Contextual Factors
Residential greenery	Air pollution mitigation, heat reduction	↓ Mortality, ↓ CVDs, ↑ respiratory health, ↑ metabolic health, ↓ obesity, ↓ cancer risk, ↑ birth outcomes, ↑ mental health and well-being	Age (children, older adults, women) and low-SES groups; vegetation diversity; UGI connectivity and clustering
Urban vegetation	Background air pollution reduction, UHI mitigation	↓ Mortality, ↓ CVDs, ↑ respiratory health, ↓ obesity, ↑ life satisfaction	Urban density; low-SES areas; extreme air pollution; UGI clustering and connectivity
Urban trees	Heat reduction, air pollution removal	↓ Mortality, ↓ CVDs, ↑ respiratory health, ↑ psychological restoration	Age (older adults) and heat-vulnerable groups; allergenic species; canopy continuity; crown volume
School greenery	Air pollution mitigation, heat reduction	↓ CVDs, ↑ respiratory and metabolic health, ↓ obesity, ↑ cognition and visual health, ↑ well-being	Children in low-SES households; allergenic species
Urban forest	Air pollution removal, cooling	↑ Respiratory health, ↓ CVDs, ↓ mortality, ↑ stress recovery	Distance to; canopy depth; stand density
Urban parks	Thermal comfort, air pollution mitigation	↑ Happiness, ↑ stress recovery, partial respiratory and CVD benefits	Age (younger adults); extreme air pollution; semi-open urban layouts; availability of shading
Green roofs and walls	Indoor cooling, thermal comfort	↓ Heat-related mortality, ↓ stress, ↑ positive emotions	Local climate; coverage density; integration with other UGI
Green streets	Heat reduction, pollution mitigation	↓ CVD mortality, ↑ respiratory health, ↑ thermal comfort, ↑ emotional recovery	Age (women); allergenic species; tree line continuity; wind/solar orientation
Grassland	Air pollution mitigation, mild cooling	↑ Physical health, ↑ sleep quality	Species diversity
Community gardens	Air pollution mitigation, heat reduction	↑ Respiratory health, ↓ diabetes risk, ↑ mood and well-being	Extreme air pollution; other shading; availability of vertical greening

## Data Availability

No new data was created or analyzed in this study. Data sharing is not applicable to this article.

## Supplementary Materials

The following supporting information can be downloaded at https://www.mdpi.com/article/10.3390/ijerph22121842/s1, Supplementary Material—Detailed search string; Supplementary Table S1—Included articles reporting ten UGI types, climate adaptation pathway, and associated health co-benefit. Reference [230] is cited in Supplementary Materials.
