# Supplementary material for: Climate, Health, and Urban Green Infrastructure: The Evidence Base and Implications for Urban Policy and Spatial Planning
Source: Int J Environ Res Public Health. 2025 Dec 9;22(12):1842. doi: 10.3390/ijerph22121842 (PMC12732387; doi:10.3390/ijerph22121842)
Supplement: Supplementary file 1 [file ijerph-22-01842-s001.zip › Supplementary Material - Detailed Search String.pdf]

Supplementary Material 1: Detailed Search String

Finally, the following search string was used for the literature search:

(title or abstract or keyword) contains

("green infrastructure" OR "green urban infrastructure" OR "nature-based solutions" OR "urban green*" OR "green spaces" OR "greenspaces" OR "green patches" OR "gardens" OR "parks" OR "greenways" OR "green streets" OR "green movements" OR "permeable pavements" OR "green roofs" OR "green walls" OR "green facades" OR "vertical farming" OR "urban agriculture" OR "urban farming" OR "urban vegetation" OR "green belts" OR "greenbelts" OR "green buffer" OR "grasslands" OR "lawns" OR "urban forests" OR "urban forestry" OR "woodlands" OR "wetlands" OR "urban trees" OR "street trees")

AND

("climate adaptation" OR "climate change" OR "climate resilience" OR "climate crisis" OR "climate benefit" OR "urban heat islands" OR "UHI" OR "urban cool islands" OR "UCI" OR "urban cooling" OR "cooling effect" OR "heat waves" OR "heat reduction" OR "outdoor thermal comfort" OR "air temperature" OR "extreme temperature" OR "land surface temperature" OR "shade" OR "shading" OR "flood" OR "flooding" OR "stormwater" OR "rainwater" OR "urban runoff" OR "rainfall" OR "snowfall" OR "air quality" OR "air pollution" OR "air pollutant" OR "air purification" OR "air cleaning" OR "carbon sequestration" OR "CO2" OR "PM2.5" OR "PM10" OR "NO2" OR "extreme weather" OR "wildfire" OR "storms" OR "drought")

AND

("human health" OR "population health" OR "public health" OR "health outcomes" OR "health effects" OR "health assessments" OR "health benefits" OR "health gains" OR "health costs" OR "health impacts" OR "health hazards" OR "health exposure" OR "physical health" OR "obesity" OR "blood pressure" OR "body mass index" OR "BMI" OR "cancer" OR "injury" OR "disease_" OR "mental health" OR "psychological health" OR "anxiety" OR "depression" OR "mood" OR "loneliness" OR "wellbeing" OR "happiness" OR "mortality" OR "life expectancy" OR "morbidity" OR "death" OR "hospital admissions" OR "planetary health" OR "one health" OR "eco-health")
