# Supplementary material for: Climate, Health, and Urban Green Infrastructure: The Evidence Base and Implications for Urban Policy and Spatial Planning
Source: Int J Environ Res Public Health. 2025 Dec 9;22(12):1842. doi: 10.3390/ijerph22121842 (PMC12732387; doi:10.3390/ijerph22121842)
Supplement: Supplementary file 1 [file ijerph-22-01842-s001.zip › Supplementary Table.pdf]

Table S1- Included Articles Reporting Ten Types of UGI, Climate Adaptation Pathway, and associated Health Co-benefits

GI Types	*Health aspects*	*Climate adaptation benefits*	*Health co-benefits evidence reported*	*Reference*
**Residential greenery**	Mortality (10 studies)	Reducing exposure to air pollutants such as PM2.5, PM_{10}, and NO_2.	Lower non-accidental and cause-specific mortality.	(Bereziartua et al., 2022; Ji et al., 2020; Klompmaker et al., 2021; Orioli et al., 2019; Roscoe et al., 2022; Vienneau et al., 2017; Wan et al., 2022; Yuan et al., 2023; Y. Zhang et al., 2025)
			The protective effects are particularly pronounced among young people and women.	(Bereziartua et al., 2022; Vienneau et al., 2017; Yuan et al., 2023)
		Mitigating extreme high temperatures	Reduce heat-related mortality among elderly individuals.	(Qiu et al., 2021)
			The protective effect is more evident in warmer regions.	(Qiu et al., 2021)
	Cardiovascular diseases (CVDs) (14 studies)	Reducing exposure to air pollutants—such as $PM_{2.5}$, PM_{10}, NO_2, O_3, and SO_2	Reduced risk of stroke.	(Avellaneda-Gómez et al., 2022; C. Liu et al., 2023)
			Lower blood pressure.	(W.-W. Bao, Jiang, et al., 2024; Chien et al., 2022; B. Huang et al., 2021; Mei et al., 2022; H. Sun et al., 2025)
			Decreased coronary atherosclerosis.	(H.-B. Hu et al., 2022; Pan et al., 2024; Riggs et al., 2021; K. Yu et al., 2023)
			Reduced incidence of ischaemic heart disease.	(T. Li et al., 2022)
			Providing the greatest protective effect for women and younger age groups (under 65 years).	(C. Liu et al., 2023; K. Yu et al., 2023)
			The aggregation index being most effective in reducing air pollution.	(Pan et al., 2024)
		Lowering high nighttime land surface temperatures	Reducing stroke risk.	(J. Bao et al., 2021; Pan et al., 2024)
			Particularly among women and the elderly.	(J. Bao et al., 2021)
			Patch density is more efficient at reducing heatwaves	(Pan et al., 2024)
		Mitigating exposure to low temperatures	Reducing cardiovascular morbidity.	(G. Wang et al., 2023)

	Respiratory health (14 studies)	Mitigating air pollutants such as $PM_{2.5}$, NO_2, NO_x, and O_3	Reduced respiratory mortality.	(S. Sun et al., 2020)
			Lowering risk of asthma.	(Alcock et al., 2017; P.-P. Dong et al., 2025; Y. Hu et al., 2023; Kim & Ahn, 2021)
			Reduced hospitalization for breathing problems.	(Damasceno da Silva et al., 2025; Xu et al., 2025)
			Improved lung function.	(Fuertes et al., 2020; W. Yu et al., 2023)
			Increased tuberculosis treatment success rates.	(F. Zhou et al., 2025)
			Dense or clustered tree patches and small community green spaces may be more effective.	(Kim & Ahn, 2021)
		The presence of allergenic plant species and the exacerbating effects of elevated levels of air pollutants	Increased risk of asthma.	(Y. Dong et al., 2021; Hsieh et al., 2019)
			Increased risk of other respiratory symptoms, such as dry cough at night and rhinitis.	(P.-P. Dong et al., 2025; L. Yang et al., 2023)
			Declining lung function.	(Markevych et al., 2023)
			Increasing plant biodiversity is a recognised mitigation strategy.	(Y. Dong et al., 2021; Hsieh et al., 2019; Y. Hu et al., 2023)
	Metabolic health (14 studies)	Mitigating air pollutants such as $PM_{2.5}$, NO_2, and O_3.	Reduced risk of diabetes.	(Cui et al., 2024; K. Hu et al., 2023; Thiering et al., 2016; T. Yang et al., 2023)
			Reduced risk of gestational diabetes mellitus (GDM).	(J. Liao, Chen, et al., 2019; Z. Yu et al., 2023; T. Zhang et al., 2025)
			Reduced Metabolic Syndrome (MetS)	(L. Liu et al., 2022; Odebeatu et al., 2025; Paoin et al., 2023; Y. Xiao et al., 2023)
			Reduced fatty liver disease and abnormal blood lipid levels.	(L. Chen et al., 2023; M. Liu et al., 2023; Torres Toda et al., 2022)
			Extremely high levels of air pollutants can significantly reduce the protective effects on various metabolic health outcomes.	(Paoin et al., 2023; T. Yang et al., 2023)
	Obesity (9 studies)	Reducing exposure to air pollutants PM1,	Reduced risk of obesity.	(Bloemsma et al., 2019; De Ryck et al., 2024; W.-Z. Huang et al., 2020; Nguyen

		PM2.5, PM10, NO2, and O3		Thi Khanh et al., 2025; O'Callaghan-Gordo et al., 2020; Persson et al., 2018; X. Xiao et al., 2021; B. Yu et al., 2024; S. Zhou et al., 2022)
			Particularly for women and individuals from lower socioeconomic groups.	(W.-Z. Huang et al., 2020; O'Callaghan-Gordo et al., 2020; Persson et al., 2018; X. Xiao et al., 2021)
	Cancer (7 studies)	Reduced exposure to particulate matter	Reduced risk of lung cancer, especially pronounced among men.	(Y.-J. Huang et al., 2022; K. Liu et al., 2025; Rodriguez-Loureiro et al., 2022; W. Wu et al., 2024)
			Reduced oral cavity cancer, pharyngeal cancer, and non-melanoma skin cancer.	(Datzmann et al., 2018)
			Reduced breast cancer.	(K. Liu et al., 2025; Odebeatu et al., 2024; Rodriguez-Loureiro et al., 2022) (O'Callaghan-Gordo et al., 2018)
	Birth outcome (6 studies)	Reducing maternal exposure to air pollutants such as $PM_{2.5}$, NO_2, and O_3	Reduced risk of preterm birth and improving neonatal birth weight.	(Akaraci et al., 2022; Y. Sun et al., 2020; D. Wang et al., 2025; Y. Yu et al., 2024)
			Mitigate the risk of infantile atopic dermatitis (AD).	(Lee et al., 2018)
		Reducing heat exposure during pregnancy	Reduced risk of miscarriage.	(X. Sun et al., 2020)
	Other physical health (12 studies)	Increased illumination, reduced heat stress index, elevated levels of negative oxygen ions, and reduced exposure to $PM_{2.5}$	Improving general physical health.	(Fang et al., 2021; B. Huang et al., 2022)
		Reduced exposure to air pollutants such as $PM_{2.5}$, PM_{10}, and NO_2	Reduced incidence of neurodegenerative diseases—such as Parkinson's disease (PD).	(Feng et al., 2024; Z. Yu et al., 2021; Zhu et al., 2023)
			Improved musculoskeletal health.	(Jiang et al., 2022; X. Zhao et al., 2025)
			Reduced risk of visual impairment in children through mitigation of PM_{10}	(W.-W. Bao, Zhao, et al., 2024)

			Lowering risk of frailty in elderly populations.	(X. Guo et al., 2024)
			Reduced prevalence of chronic kidney disease (CKD).	(Liang et al., 2022)
			Decreased risk of severe liver disease.	(Ye et al., 2023)
			Lowering prevalence of thyroid nodules.	(C. Liu et al., 2024)
	Mental health (13 studies)	Reducing exposure to traffic-related air pollutants such as $PM_{2.5}$ and NO_2	A lower risk of attention deficit hyperactivity disorder (ADHD).	(Yuchi et al., 2022)
			Improvements in attention, concentration, and numerical reasoning in newborns.	(Asta et al., 2021)
			Enhanced early childhood mental and psychomotor development indices.	(J. Liao, Zhang, et al., 2019)
			Improved working memory.	(Dadvand et al., 2015)
			A reduced risk of autism spectrum disorder (ASD).	(Pagalan et al., 2022)
		Reducing perceived air pollution	Reducing anxiety and depression, particularly in areas with relatively low baseline pollution levels.	(Bakhtsiyarava et al., 2024; H. Li et al., 2022; Motoc et al., 2025; Mouly et al., 2023; Triebner et al., 2022; Xue et al., 2025; C. Yang et al., 2022; Yi et al., 2025)
	Well-being (3 studies)	Improved perceived air quality and enhanced social cohesion	Improving happiness, life satisfaction, and reduced stress.	(D. Huang et al., 2023; Y. Liu et al., 2022; R. Wang et al., 2021)
**Urban vegetation**	Mortality (11 studies)	Mitigating urban heat island (UHI) effect	Reducing heat-related mortality.	(Burkart et al., 2016; Choi et al., 2022; T. N. Dang et al., 2018; Kalkstein et al., 2022; C.-C. Li et al., 2023; Pascal et al., 2021; Schinasi et al., 2023; Son et al., 2016; J. Song et al., 2024)
			Particularly beneficial for the elderly.	(Burkart et al., 2016; Son et al., 2016)
			Especially in densely populated areas vulnerable to high temperatures.	(Pascal et al., 2021)
			Increasing tree canopy cover, permeable surfaces, and urban surface albedo.	(Kalkstein et al., 2022; Pascal et al., 2021)
			Spatially clustering green elements.	(Schinasi et al., 2023)

		Mitigating long-term exposure to PM2.5, PM10	Lower mortality and higher life expectancy.	(de Keijzer et al., 2017; C.-C. Li et al., 2023; Tang et al., 2025)
			Especially evident in areas with lower socioeconomic status.	(de Keijzer et al., 2017)
	Cardiovascular diseases (CVDs) (6 studies)	Mitigating air pollution PM2.5, PM10	Reduce hypertension.	(Q. Chen et al., 2022; W. Zhou, Wang, Li, et al., 2023)
			Reduce hospital admissions for CVDs.	(Heo & Bell, 2019)
			Reduce CVD-related mortality.	(Shen & Lung, 2016; C. Zhao et al., 2023)
			Especially for younger elderly groups (65-84 yrs).	(Heo & Bell, 2019)
		Heat reduction	Lowering the risk of hypertension.	(W. Zhou, Wang, Li, et al., 2023)
			Reduce heat stroke.	(Heo et al., 2021)
			Reduce CVD-related mortality.	(Shen & Lung, 2016)
			Especially for older adults (≥85 yrs)—particularly women.	(Heo & Bell, 2019)
	Respiratory health (5 studies)	Mitigation of air pollutants such as $PM_{2.5}$ and PM_{10}	Reduce hospital admissions for respiratory diseases.	(Heo & Bell, 2019)
			Lowering respiratory-related mortality.	(Jaafari et al., 2020)
			Decrease transmission and mortality associated with COVID-19.	(Meo et al., 2021)
			Total green area (CA) and the cohesion index (COHESION) were identified as key positive influencing factors, whereas greater dispersion (TE) negatively affects the former effectiveness.	(Jaafari et al., 2020)
		Increased pollen exposure or elevated air pollution levels	Negatively impact childhood asthma and allergic rhinitis.	(Kwon et al., 2019; Malamardi et al., 2022)
	Obesity (2 studies)	Mitigation of air pollution $PM_{2.5}$, PM_{10}, SO_2, NO_2, CO, and O_3	Reduced obesity rate.	(G. Li et al., 2022; W. Zhou, Wang, Kadier, et al., 2023)
			When pollution levels are excessively high or when multiple pollutants are present simultaneously, the protective effect is	(G. Li et al., 2022; W. Zhou, Wang, Kadier, et al., 2023)

			significantly diminished.	
	Cancer (2 studies)	Reduced exposure to PM2.5	Reduction of lung cancer.	(Pang et al., 2024)
			The protective effect exhibited a spatial spillover effect (20%).	(Pang et al., 2024)
		Reduced long-term exposure to PM10	Reduction of breast cancer.	(Terre-Torras et al., 2022)
	General physical health (3 studies)	Mitigating air pollution PM2.5, PM10, SO2	Improving general regional residents' health and self-reported physical health.	(Fu et al., 2024; L. Liao & Du, 2022)
			Especially for regions with lower baseline air pollution level.	(Fu et al., 2024)
		Mitigating air pollutants O3	Protect against oxidative stress.	(Qi et al., 2024)
	Mental health (2 studies)	Reduced exposure to air pollution	Reduced risk of anxiety.	(Shen, 2025)
			Improved cognitive function in older adults.	(A. Chen et al., 2025)
		Heat reduction	Reduced risk of anxiety.	(Shen, 2025)
			Highly dispersed green patches were linked to higher anxiety-disorder prevalence.	(Shen, 2025)
	Well-being (2 studies)	Air pollution mitigation	Enhanced life satisfaction.	(L. Wu & Chen, 2023)
			Highly dispersed green patches were linked to reduced life satisfaction.	(L. Wu & Chen, 2023)
		Reduction of water-logging risk	Increase residents' happiness.	(X. Yang et al., 2025)
**School greenery**	Cardiovascular diseases (CVDs) (2 studies)	Lowering levels of air pollutants such as NO_2, PM_{10}, and PM_1	Reduce the risk of CVDs (especially hypertension) in children.	(W.-W. Bao, Jiang, et al., 2024; X. Xiao et al., 2020)
			Particularly pronounced among children from low-income families.	(W.-W. Bao, Jiang, et al., 2024)
	Respiratory health (3 studies)	Mitigation of air pollutants such as $PM_{2.5}$, NO_2, and O_3	Lowering the risk of adverse respiratory symptoms and asthma.	(P.-P. Dong et al., 2025; L. Yang et al., 2023; H. Yu et al., 2021)
	Metabolic health	Lowering levels of air	Lower prevalence of metabolic health risks,	(L.-X. Hu et al., 2023)

	(1 study)	pollution ($PM_{2.5}$, NO_2)	particularly evident among children with lower levels of parental education.	
	Obesity<br>(1 study)	Lowering levels of air pollution ($PM_{2.5}$, NO_2)	Reduced rates of overweight and obesity in children.	(W.-W. Bao et al., 2021)
	Other physical health<br>(1 study)	Mitigating air pollutant PM10	Benefit children's vision (e.g., visual acuity).	(W.-W. Bao, Zhao, et al., 2024)
	Mental health<br>(3 studies)	Reducing exposure to traffic-related air pollution	Positively influences children's cognitive development.	(Dadvand et al., 2015; Requia et al., 2022)
		Reducing exposure to NO2, O3	Improve general mental health.	(J. Dang et al., 2025)

**Urban trees**	Mortality<br>(11 studies)	Mitigating UHI effect	Reducing heat-related mortality and morbidity.	(Chi et al., 2025; Iungman et al., 2023; McDonald et al., 2020, 2024; Pascal et al., 2021; Sadeghi et al., 2022; Sheridan et al., 2024; Sinha et al., 2021; Taylor et al., 2024)
			More pronounced among older adults.	(Sinha et al., 2021)
			The cooling benefits are particularly significant in cities with low baseline tree coverage and significant UHI effects.	(McDonald et al., 2024)
		Mitigating air pollutants PM2.5, PM10, NO2, and O3	Reduce non-accidental and case-specific mortality.	(Chi et al., 2025; Sicard et al., 2025; Vranken et al., 2023)
	Respiratory health<br>(3 studies)	Mitigation of air pollutants such as $PM_{2.5}$, NO_2, O_3, and SO_2	Reducing risks of general respiratory problem and asthma.	(Alcock et al., 2017; P. Chen & Hanlon, 2025; Kim & Ahn, 2021)
			The protective effect being more pronounced at higher levels of air pollution.	(Alcock et al., 2017)
			Creating dense or clustered tree patches may be more effective in reducing asthma incidence than larger green spaces.	(Kim & Ahn, 2021)

	Other physical health (1 study)	Reducing air temperature	Improving sleep quality.	(Beele et al., 2024)
**Urban forests**	Cardiovascular diseases (CVDs) (2 studies)	Reduced exposure to air pollution and noise	Temporary improvements in CVD risk factors.	(Lanki et al., 2017)
		Mitigating extreme high temperature	Reduced risk of CVD-related mortality.	(K. Hu et al., 2024)
	Mortality (1 study)	Mitigating air pollutants $PM_{2.5}$ and PM_{10}	Logged fewer deaths from all causes, particularly heart- and lung-related illnesses.	(S. Liu et al., 2024)
	Respiratory health (4 studies)	Mitigating air pollution PM2.5, PM10, NO2, O3	Reducing the risk of various respiratory health problems, particularly for urban forest with higher tree cover.	(Aerts et al., 2020; e Almeida et al., 2020; Jeong et al., 2025; Nowak et al., 2018)
	Well-being (3 studies)	Reducing exposure to air pollutants $PM_{2.5}$ and PM_{10}	Alleviating mood disturbances and promoting stress recovery	(Ashraf et al., 2024; J. Zhang et al., 2023)
		Regulating thermal comfort	Improving perceived stress recovery and reducing negative emotions.	(S. Song et al., 2024)
**Urban parks**	Cardiovascular diseases (CVDs) (1 study)	Reduction of PM_{10}	Improving cardiovascular physiology	(Lanki et al., 2017)
	Respiratory health (1 study)	Mitigating $PM_{2.5}$	Benefit respiratory health	(K. Li et al., 2024)
	Well-being (4 studies)	Air pollution mitigation	Stimulating happiness	(Hou et al., 2024; D. Huang et al., 2023)
			Semi-open or semi-enclosed green spaces appear to offer greater psychological restorative benefits compared to fully open or closed green spaces.	(Hou et al., 2024).
		Regulation of thermal comfort	Evoking positive emotions, and promoting stress recovery	(W. Guo et al., 2022; Niu et al., 2023)
			Tree canopies were identified as the most effective	(W. Guo et al., 2022)

			cooling element in urban parks.	
			Younger individuals were found to be more sensitive to changes in thermal comfort than older adults, making their well-being more susceptible to environmental variations	(W. Guo et al., 2022; Niu et al., 2023)
**Green roofs**	Mortality (3 studies)	Lowering indoor temperatures during heatwaves	Reducing heat-related mortality.	(He et al., 2020; Marvuglia et al., 2020; Sadeghi et al., 2022)
			The effectiveness of green roofs influenced by local temperature thresholds for heatwave mortality.	(Marvuglia et al., 2020; Sadeghi et al., 2022)
	Mental health (1 study)	Heat reduction	Reduce the risk of depression.	(Sung et al., 2025)
**Green walls**	Mental health and well-being (1 study)	Regulating thermal comfort	Boosting positive emotions, reduce stress, and slightly improve cognitive abilities.	(Ma et al., 2024).
**Green streets**	Cardiovascular diseases (CVDs) (2 studies)	Reducing heat exposure	Reducing CVDs mortality.	(K. Hu et al., 2024)
		Mitigating air pollution PM2.5	Reducing CVDs morbidity. The protective effects being especially significant for women.	(R. Wang et al., 2022)
	Respiratory health (1 study)	Lowering exposure to $PM_{2.5}$	Reducing the respiratory health burden, especially street trees.	(Lai & Kontokosta, 2019)
			The presence of allergenic tree species, such as red maple, northern red oak, and American linden, has been associated with increased asthma hospitalisations.	(Lai & Kontokosta, 2019)
	Well-being (2 studies)	Provide more effective shading and cooling, higher relative humidity, and lower wind speeds	Enhanced recovery and feelings of vigour. Especially areas with high tree canopies.	(Elsadek et al., 2019; Klemm et al., 2015)
**Grasslands**	General physical health (2 studies)	Mitigating air pollution and heat reduction	Provide general physical health benefits (e.g, morbidity, sleep quality)	(Beele et al., 2024; Vranken et al., 2023)

	Respiratory health (2 studies)	Increased pollen exposure	Potential negative effects of grasslands for respiratory health, such as asthma rates.	(Aerts et al., 2020; P. Chen & Hanlon, 2025)
			Living in areas with high grassland coverage—such as permanent grasslands, hay meadows, and lawns—may have negative impacts on children's respiratory health.	(Aerts et al., 2020)
	Well-being (1 study)	Enhancing thermal comfort	Improve participants' perceived recovery and reduce negative emotions.<br>Slightly less effective than the urban forest in reducing anger.	(S. Song et al., 2024)
Community gardens	Respiratory health (2 studies)	Mitigating air pollutants such as NO_2, $PM_{2.5}$, PM_{10}, and SO_2	Mitigating respiratory health risks.	(Aerts et al., 2020; Alcock et al., 2017)
			These protective effects were weakened or absent at higher pollution levels.	(Alcock et al., 2017)
	Well-being (1 study)	Mitigating UHI effect and improving thermal comfort	Positively influences mood.<br>Particularly those incorporating shaded areas, such as trees and shelters with climbing plants.	(Mosca et al., 2021)
Private gardens	Metabolic health (1 study)	Mitigation of air pollutants such as $PM_{2.5}$ and NO_2	Reduce the incidence of diabetes.	(T. Yang et al., 2023)
	General physical health (1 study)	Mitigation of air pollutants such as $PM_{2.5}$ and NO_2	Lower the risk of various air pollution-related health outcomes.	(Chang et al., 2024)

Reference list:

Aerts, R., Dujardin, S., Nemery, B., Van Nieuwenhuyse, A., Van Orshoven, J., Aerts, J.-M., Somers, B., Hendrickx, M., Bruffaerts, N., Bauwelinck, M., Casas, L., Demoury, C., Plusquin, M., & Nawrot, T. S. (2020). Residential green space and medication sales for childhood asthma: A longitudinal ecological study in Belgium. *Environmental Research*, *189*, 109914. https://doi.org/10.1016/j.envres.2020.109914

Akaraci, S., Feng, X., Suesse, T., Jalaludin, B., & Astell-Burt, T. (2022). Associations between green space, air pollution and birthweight in Sydney Metropolitan Area, Australia. *Urban Forestry & Urban Greening*, *76*, 127726. https://doi.org/10.1016/j.ufug.2022.127726

Alcock, I., White, M., Cherrie, M., Wheeler, B., Taylor, J., McInnes, R., Otte im Kampe, E., Vardoulakis, S., Sarran, C., Soyiri, I., & Fleming, L. (2017). Land cover and air pollution are associated with asthma hospitalisations: A cross-sectional study. *Environment International*, *109*, 29–41.

https://doi.org/10.1016/j.envint.2017.08.009
Ashraf, K., Park, Y. M., Browning, M. H. E. M., Wang, J., Wang, R., & Lee, K. (2024). Examining the joint effect of air pollution and green spaces on stress levels in South Korea: Using machine learning techniques. *International Journal of Digital Earth*, *17*(1), 2372321. https://doi.org/10.1080/17538947.2024.2372321
Asta, F., Michelozzi, P., Cesaroni, G., De Sario, M., Davoli, M., & Porta, D. (2021). Green spaces and cognitive development at age 7 years in a rome birth cohort: The mediating role of nitrogen dioxide. *Environmental Research*, *196*, 110358. https://doi.org/10.1016/j.envres.2020.110358
Avellaneda-Gómez, C., Vivanco-Hidalgo, R. M., Olmos, S., Lazcano, U., Valentin, A., Milà, C., Ambrós, A., Roquer, J., & Tonne, C. (2022). Air pollution and surrounding greenness in relation to ischemic stroke: A population-based cohort study. *Environment International*, *161*, 107147. https://doi.org/10.1016/j.envint.2022.107147
Bakhtsiyarava, M., Ju, Y., Moran, M., Rodríguez, D. A., Dronova, I., Delclòs-Alió, X., Moore, K., Castillo-Riquelme, M., & Anza-Ramirez, C. (2024). Associations between urban greenspace and depressive symptoms in Mexico's cities using different greenspace metrics. *Applied Geography*, *164*, 103219. https://doi.org/10.1016/j.apgeog.2024.103219
Bao, J., Cao, Z., Huang, C., Lei, L., Yang, Y., Peng, J., & Shi, X. (2021). Modification effect of urban landscape characteristics on the association between heat and stroke morbidity: A small-scale intra-urban study in Shenzhen, China. *Science of The Total Environment*, *786*, 147223. https://doi.org/10.1016/j.scitotenv.2021.147223
Bao, W.-W., Jiang, N., Zhao, Y., Yang, B., Chen, G., Pu, Y., Ma, H., Liang, J., Xiao, X., Guo, Y., Dong, G., & Chen, Y. (2024). Urban greenspaces and child blood pressure in China: Evidence from a large population-based cohort study. *Environmental Research*, *244*, 117943. https://doi.org/10.1016/j.envres.2023.117943
Bao, W.-W., Yang, B.-Y., Zou, Z.-Y., Ma, J., Jing, J., Wang, H.-J., Luo, J.-Y., Zhang, X., Luo, C.-Y., Wang, H., Zhao, H.-P., Pan, D.-H., Gui, Z.-H., Zhang, J.-S., Guo, Y.-M., Ma, Y.-H., Dong, G.-H., & Chen, Y.-J. (2021). Greenness surrounding schools and adiposity in children and adolescents: Findings from a national population-based study in China. *Environmental Research*, *192*, 110289. https://doi.org/10.1016/j.envres.2020.110289
Bao, W.-W., Zhao, Y., Dadvand, P., Jiang, N., Chen, G., Yang, B., Huang, W., Xiao, X., Liang, J., Chen, Y., Huang, S., Pu, X., Huang, S., Lin, H., Guo, Y., Dong, G., & Chen, Y. (2024). Urban greenspace and visual acuity in schoolchildren: A large prospective cohort study in China. *Environment International*, *184*, 108423. https://doi.org/10.1016/j.envint.2024.108423
Beele, E., Aerts, R., Reyniers, M., & Somers, B. (2024). Urban green space, human heat perception and sleep quality: A repeated cross-sectional study. *Environmental Research*, *263*, 120129. https://doi.org/10.1016/j.envres.2024.120129
Bereziartua, A., Chen, J., de Hoogh, K., Rodopoulou, S., Andersen, Z. J., Bellander, T., Brandt, J., Fecht, D., Forastiere, F., Gulliver, J., Hertel, O., Hoffmann, B., Arthur Hvidtfeldt, U., Verschuren, W. M. M., Jöckel, K.-H., Jørgensen, J. T., Katsouyanni, K., Ketzel, M., Hjertager Krog, N., … Hoek, G. (2022). Exposure to surrounding greenness and natural-cause and cause-specific mortality in the ELAPSE pooled cohort. *Environment International*, *166*, 107341. https://doi.org/10.1016/j.envint.2022.107341
Bloemsma, L. D., Wijga, A. H., Klompmaker, J. O., Janssen, N. A. H., Smit, H. A., Koppelman, G. H., Brunekreef, B., Lebret, E., Hoek, G., & Gehring, U. (2019). The associations of air pollution, traffic noise and green space with overweight throughout childhood: The PIAMA birth cohort study. *Environmental Research*, *169*, 348–356. https://doi.org/10.1016/j.envres.2018.11.026

Burkart, K., Meier, F., Schneider, A., Breitner, S., Canário, P., Alcoforado, M. J., Scherer, D., & Endlicher, W. (2016). Modification of Heat-Related Mortality in an Elderly Urban Population by Vegetation (Urban Green) and Proximity to Water (Urban Blue): Evidence from Lisbon, Portugal. *Environmental Health Perspectives*, *124*(7), 927–934. https://doi.org/10.1289/ehp.1409529

Chang, Y. H., Chen, T.-H., Chung, H.-Y., Hsiao, H.-Y., Tseng, P.-C., Wang, Y.-C., Candice Lung, S.-C., Su, H.-J., & Tsay, Y.-S. (2024). The health risk reduction of PM2.5 via a green curtain system in Taiwan. *Building and Environment*, *255*, 111459. https://doi.org/10.1016/j.buildenv.2024.111459

Chen, A., Cao, K., Liu, Y., Li, S. L., Chen, H., & Chiu, M. Y.-L. (2025). Nexus Between Exposure to Natural Outdoor Environments and Cognitive Competence among Older Adults in China. *Annals of the American Association of Geographers*, *115*(5), 1146–1164. https://doi.org/10.1080/24694452.2024.2380889

Chen, L., Jia, Y., Guo, Y., Chen, G., Ciren, Z., Chen, H., Duoji, Z., Xu, J., Yang, T., Xu, H., Feng, S., Jiang, Y., Guo, B., Meng, Q., & Zhao, X. (2023). Residential greenness associated with decreased risk of metabolic- dysfunction-associated fatty liver disease: Evidence from a large population-based epidemiological study. *Ecotoxicology and Environmental Safety*, *249*, 114338. https://doi.org/10.1016/j.ecoenv.2022.114338

Chen, P., & Hanlon, B. (2025). Urban green space, respiratory health and rising temperatures: An examination of the complex relationship between green space and adult asthma across racialized neighborhoods in Los Angeles County. *Landscape and Urban Planning*, *258*, 105320. https://doi.org/10.1016/j.landurbplan.2025.105320

Chen, Q., Ma, X., Geng, Y., Liao, J., & Ma, L. (2022). Association between smoking and hypertension under different PM2.5 and green space exposure: A nationwide cross-sectional study. *Frontiers in Public Health*, *10*. https://doi.org/10.3389/fpubh.2022.1026648

Chi, D., Manoli, G., Lin, B., Aerts, R., Yang, J., Hahs, A., Richards, D., Meili, N., Zhu, Y., Qiu, Y., Wang, J., Burlando, P., Fatichi, S., & Tan, P. Y. (2025). Residential tree canopy configuration and mortality in 6 million Swiss adults: A longitudinal study. *The Lancet Planetary Health*, *9*(3), e186–e195. https://doi.org/10.1016/S2542-5196(25)00022-1

Chien, J.-W., Wu, C., & Chan, C.-C. (2022). The association of hypertension and prehypertension with greenness and PM2.5 in urban environment. *Science of The Total Environment*, *821*, 153526. https://doi.org/10.1016/j.scitotenv.2022.153526

Choi, H. M., Lee, W., Roye, D., Heo, S., Urban, A., Entezari, A., Vicedo-Cabrera, A. M., Zanobetti, A., Gasparrini, A., Analitis, A., Tobias, A., Armstrong, B., Forsberg, B., Íñiguez, C., Åström, C., Indermitte, E., Lavigne, E., Mayvaneh, F., Acquaotta, F., … Bell, M. L. (2022). Effect modification of greenness on the association between heat and mortality: A multi-city multi-country study. *eBioMedicine*, *84*. https://doi.org/10.1016/j.ebiom.2022.104251

Cui, Z., Pan, R., Liu, J., Yi, W., Huang, Y., Li, M., Zhang, Z., Kuang, L., Liu, L., Wei, N., Song, R., Yuan, J., Li, X., Yi, X., Song, J., & Su, H. (2024). Green space and its types can attenuate the associations of PM2.5 and its components with prediabetes and diabetes——A multicenter cross-sectional study from eastern China. *Environmental Research*, *245*, 117997. https://doi.org/10.1016/j.envres.2023.117997

Dadvand, P., Nieuwenhuijsen, M. J., Esnaola, M., Forns, J., Basagaña, X., Alvarez-Pedrerol, M., Rivas, I., López-Vicente, M., De Castro Pascual, M., Su, J., Jerrett, M., Querol, X., & Sunyer, J. (2015). Green spaces and cognitive development in primary schoolchildren. *Proceedings of the National Academy of Sciences*, *112*(26), 7937–7942. https://doi.org/10.1073/pnas.1503402112

Damasceno da Silva, R. M., Castelhano, F. J., Albino Sitoe, G. A., Hoinaski, L., Amini, H., Saldiva, P. H. N., & Requia, W. J. (2025). The mediating role of air pollution in the relationship between greenspace and cardiorespiratory admissions in Brazil. *Environmental Pollution*, *369*, 125849. https://doi.org/10.1016/j.envpol.2025.125849

Dang, J., Wang, Y., Ma, N., Cai, S., Guo, J., Liu, Y., Zhou, H., Lian, X., Shi, D., Chen, Z., Zhang, Y., Li, J., Huang, T., Zhu, G., Li, J., Ma, J., & Song, Y. (2025). The impact of long-term exposure to NO2, O3, and their oxidative potential on adolescents' mental health, and the protective role of school-based greenness. *Environment International*, *195*, 109212. https://doi.org/10.1016/j.envint.2024.109212

Dang, T. N., Van, D. Q., Kusaka, H., Seposo, X. T., & Honda, Y. (2018). Green Space and Deaths Attributable to the Urban Heat Island Effect in Ho Chi Minh City. *American Journal of Public Health*, *108*(S2), S137–S143. https://doi.org/10.2105/AJPH.2017.304123

Datzmann, T., Markevych, I., Trautmann, F., Heinrich, J., Schmitt, J., & Tesch, F. (2018). Outdoor air pollution, green space, and cancer incidence in Saxony: A semi-individual cohort study. *BMC Public Health*, *18*(1), 715. https://doi.org/10.1186/s12889-018-5615-2

de Keijzer, C., Agis, D., Ambrós, A., Arévalo, G., Baldasano, J. M., Bande, S., Barrera-Gómez, J., Benach, J., Cirach, M., Dadvand, P., Ghigo, S., Martinez-Solanas, È., Nieuwenhuijsen, M., Cadum, E., & Basagaña, X. (2017). The association of air pollution and greenness with mortality and life expectancy in Spain: A small-area study. *Environment International*, *99*, 170–176. https://doi.org/10.1016/j.envint.2016.11.009

De Ryck, E., Ghosh, M., Nawrot, T. S., Reimann, B., Koppen, G., Verachtert, E., Devlieger, R., Godderis, L., & Pauwels, S. (2024). Effects of exposure to environmental factors on obesity-related growth parameters and leptin (*LEP)* methylation in children. *Environmental Pollution*, *346*, 123465. https://doi.org/10.1016/j.envpol.2024.123465

Dong, P.-P., Chen, G., Wen, Y., Heinrich, J., Fuertes, E., Zhao, T., Idrose, N. S., Lin, L.-Z., Gui, Z.-H., Qin, X., Tu, H., & Dong, G.-H. (2025). Greenspace and respiratory diseases and symptoms in Chinese children: A large-scale study exploring joint associations with ozone. *Sustainable Cities and Society*, *124*, 106329. https://doi.org/10.1016/j.scs.2025.106329

Dong, Y., Liu, H., & Zheng, T. (2021). Association between Green Space Structure and the Prevalence of Asthma: A Case Study of Toronto. *International Journal of Environmental Research and Public Health*, *18*(11), Article 11. https://doi.org/10.3390/ijerph18115852

e Almeida, L. de O., Favaro, A., Raimundo-Costa, W., Anhê, A. C. B. M., Ferreira, D. C., Blanes-Vidal, V., & dos Santos Senhuk, A. P. M. (2020). Influence of urban forest on traffic air pollution and children respiratory health. *Environmental Monitoring and Assessment*, *192*(3), 175. https://doi.org/10.1007/s10661-020-8142-4

Elsadek, M., Liu, B., Lian, Z., & Xie, J. (2019). The influence of urban roadside trees and their physical environment on stress relief measures: A field experiment in Shanghai. *Urban Forestry & Urban Greening*, *42*, 51–60. https://doi.org/10.1016/j.ufug.2019.05.007

Fang, Y., Que, Q., Tu, R., Liu, Y., & Gao, W. (2021). How do landscape elements affect public health in subtropical high-density city: The pathway through the neighborhood physical environmental factors. *Building and Environment*, *206*, 108336. https://doi.org/10.1016/j.buildenv.2021.108336

Feng, Y., Li, M., Hao, X., Ma, D., Guo, M., Zuo, C., Li, S., Liang, Y., Hao, C., Wang, Z., Sun, Y., Qi, S., Sun, S., & Shi, C. (2024). Air pollution, greenspace exposure and risk of Parkinson's disease: A prospective study of 441,462 participants. *Journal of Neurology*, *271*(8), 5233–5245. https://doi.org/10.1007/s00415-024-12492-0

Fu, J., Fu, H., Zhu, C., Sun, Y., & Cao, H. (2024). Assessing the health risk impacts of urban green spaces on air pollution—Evidence from 31 China's provinces. *Ecological Indicators*, *159*, 111725. https://doi.org/10.1016/j.ecolind.2024.111725

Fuertes, E., Markevych, I., Thomas, R., Boyd, A., Granell, R., Mahmoud, O., Heinrich, J., Garcia-Aymerich, J., Roda, C., Henderson, J., & Jarvis, D. (2020). Residential greenspace and lung function up to 24 years of age: The ALSPAC birth cohort. *Environment International*, *140*, 105749. https://doi.org/10.1016/j.envint.2020.105749

Guo, W., Jiang, L., Cheng, B., Yao, Y., Wang, C., Kou, Y., Xu, S., & Xian, D. (2022). A study of subtropical park thermal comfort and its influential factors during summer. *Journal of Thermal Biology*, *109*, 103304. https://doi.org/10.1016/j.jtherbio.2022.103304

Guo, X., Su, W., Wang, X., Hu, W., Meng, J., Ahmed, M. A., Qu, G., & Sun, Y. (2024). Assessing the effects of air pollution and residential greenness on frailty in older adults: A prospective cohort study from China. *Environmental Science and Pollution Research*, *31*(6), 9091–9105. https://doi.org/10.1007/s11356-023-31741-9

He, C., He, L., Zhang, Y., Kinney, P. L., & Ma, W. (2020). Potential impacts of cool and green roofs on temperature-related mortality in the Greater Boston region. *Environmental Research Letters*, *15*(9), 094042. https://doi.org/10.1088/1748-9326/aba4c9

Heo, S., & Bell, M. L. (2019). The influence of green space on the short-term effects of particulate matter on hospitalization in the U.S. for 2000–2013. *Environmental Research*, *174*, 61–68. https://doi.org/10.1016/j.envres.2019.04.019

Heo, S., Chen, C., Kim, H., Sabath, B., Dominici, F., Warren, J. L., Di, Q., Schwartz, J., & Bell, M. L. (2021). Temporal changes in associations between high temperature and hospitalizations by greenspace: Analysis in the Medicare population in 40 U.S. northeast counties. *Environment International*, *156*, 106737. https://doi.org/10.1016/j.envint.2021.106737

Hou, J., Wang, Y., Zhang, X., Qiu, L., & Gao, T. (2024). The effect of visibility on green space recovery, perception and preference. *Trees, Forests and People*, *16*, 100538. https://doi.org/10.1016/j.tfp.2024.100538

Hsieh, C.-J., Yu, P.-Y., Tai, C.-J., Jan, R.-H., Wen, T.-H., Lin, S.-W., & Tseng, C.-C. (2019). Association between the First Occurrence of Asthma and Residential Greenness in Children and Teenagers in Taiwan. *International Journal of Environmental Research and Public Health*, *16*(12), Article 12. https://doi.org/10.3390/ijerph16122076

Hu, H.-B., Hou, Z.-H., Huang, C.-H., LaMonte, M. J., Wang, M., & Lu, B. (2022). Associations of exposure to residential green space and neighborhood walkability with coronary atherosclerosis in Chinese adults. *Environmental Pollution*, *292*, 118347. https://doi.org/10.1016/j.envpol.2021.118347

Hu, K., Wang, S., Fei, F., Song, J., Chen, F., Zhao, Q., Shen, Y., Fu, J., Zhang, Y., Cheng, J., Zhong, J., Yang, X., & Wu, J. (2024). Modifying temperature-related cardiovascular mortality through green-blue space exposure. *Environmental Science and Ecotechnology*, *20*, 100408. https://doi.org/10.1016/j.ese.2024.100408

Hu, K., Zhang, Z., Li, Y., Wang, S., Ye, T., Song, J., Zhang, Y., Wei, J., Cheng, J., Shen, Y., Pan, J., Fu, J., Qi, J., Guo, Y., Zeng, Y., & Yao, Y. (2023). Urban overall and visible greenness and diabetes among older adults in China. *Landscape and Urban Planning*, *240*, 104881. https://doi.org/10.1016/j.landurbplan.2023.104881

Hu, L.-X., Fan, S., Ma, Y., Liu, X.-X., Bao, W.-W., Guo, Y., Hu, L.-W., Chen, G., Zeng, X.-W., Zou, Z., Yang, B.-Y., Ma, J., Yang, Z., Chen, Y.-J., & Dong, G.-H. (2023). Associations between greenspace surrounding schools and lipid levels in Chinese children and teenagers. *Environmental Pollution*, *317*, 120746. https://doi.org/10.1016/j.envpol.2022.120746

Hu, Y., Chen, Y., Liu, S., Tan, J., Yu, G., Yan, C., Yin, Y., Li, S., & Tong, S. (2023). Higher greenspace exposure is associated with a decreased risk of childhood asthma in Shanghai – A megacity in China. *Ecotoxicology and Environmental Safety*, *256*, 114868. https://doi.org/10.1016/j.ecoenv.2023.114868

Huang, B., Xiao, T., Grekousis, G., Zhao, H., He, J., Dong, G., & Liu, Y. (2021). Greenness-air pollution-physical activity-hypertension association among middle-aged and older adults: Evidence from urban and rural China. *Environmental Research*, *195*, 110836.

https://doi.org/10.1016/j.envres.2021.110836

Huang, B., Yao, Z., Pearce, J. R., Feng, Z., James Browne, A., Pan, Z., & Liu, Y. (2022). Non-linear association between residential greenness and general health among old adults in China. *Landscape and Urban Planning*, *223*, 104406. https://doi.org/10.1016/j.landurbplan.2022.104406

Huang, D., Tian, M., & Yuan, L. (2023). Do objective and subjective traffic-related pollution, physical activity and nature exposure affect mental wellbeing? Evidence from Shenzhen, China. *Science of The Total Environment*, *869*, 161819. https://doi.org/10.1016/j.scitotenv.2023.161819

Huang, W.-Z., Yang, B.-Y., Yu, H.-Y., Bloom, M. S., Markevych, I., Heinrich, J., Knibbs, L. D., Leskinen, A., Dharmage, S. C., Jalaludin, B., Morawska, L., Jalava, P., Guo, Y., Lin, S., Zhou, Y., Liu, R.-Q., Feng, D., Hu, L.-W., Zeng, X.-W., … Dong, G.-H. (2020). Association between community greenness and obesity in urban-dwelling Chinese adults. *Science of The Total Environment*, *702*, 135040. https://doi.org/10.1016/j.scitotenv.2019.135040

Huang, Y.-J., Lee, P.-H., Chen, L.-C., Lin, B.-C., Lin, C., & Chan, T.-C. (2022). Relationships among green space, ambient fine particulate matter, and cancer incidence in Taiwan: A 16-year retrospective cohort study. *Environmental Research*, *212*, 113416. https://doi.org/10.1016/j.envres.2022.113416

Iungman, T., Cirach, M., Marando, F., Barboza, E. P., Khomenko, S., Masselot, P., Quijal-Zamorano, M., Mueller, N., Gasparrini, A., Urquiza, J., Heris, M., Thondoo, M., & Nieuwenhuijsen, M. (2023). Cooling cities through urban green infrastructure: A health impact assessment of European cities. *The Lancet*, *401*(10376), 577–589. https://doi.org/10.1016/S0140-6736(22)02585-5

Jaafari, S., Shabani, A. A., Moeinaddini, M., Danehkar, A., & Sakieh, Y. (2020). Applying landscape metrics and structural equation modeling to predict the effect of urban green space on air pollution and respiratory mortality in Tehran. *Environmental Monitoring and Assessment*, *192*(7), 412. https://doi.org/10.1007/s10661-020-08377-0

Jeong, J., Kim, C., Choi, S., Sou, H.-D., & Park, C.-R. (2025). Long-Term Greenness Effects of Urban Forests to Reduce PM10 Concentration: Does the Impact Benefit the Population Vulnerable to Asthma? *International Journal of Environmental Research and Public Health*, *22*(2), Article 2. https://doi.org/10.3390/ijerph22020167

Ji, J. S., Zhu, A., Lv, Y., & Shi, X. (2020). Interaction between residential greenness and air pollution mortality: Analysis of the Chinese Longitudinal Healthy Longevity Survey. *The Lancet Planetary Health*, *4*(3), e107–e115. https://doi.org/10.1016/S2542-5196(20)30027-9

Jiang, Y., Kang Zhuo, B. M., Guo, B., Zeng, P.-B., Guo, Y.-M., Chen, G.-B., Wei, J., He, R.-F., Li, Z.-F., Zhang, X.-H., Wang, Z.-Y., Li, X., Wang, L., Zeng, C.-M., Chen, L., Xiao, X., & Zhao, X. (2022). Living near greenness is associated with higher bone strength: A large cross-sectional epidemiological study in China. *Science of The Total Environment*, *831*, 155393. https://doi.org/10.1016/j.scitotenv.2022.155393

Kalkstein, L. S., Eisenman, D. P., de Guzman, E. B., & Sailor, D. J. (2022). Increasing trees and high-albedo surfaces decreases heat impacts and mortality in Los Angeles, CA. *International Journal of Biometeorology*, *66*(5), 911–925. https://doi.org/10.1007/s00484-022-02248-8

Kim, D., & Ahn, Y. (2021). The Contribution of Neighborhood Tree and Greenspace to Asthma Emergency Room Visits: An Application of Advanced Spatial Data in Los Angeles County. *International Journal of Environmental Research and Public Health*, *18*(7), Article 7. https://doi.org/10.3390/ijerph18073487

Klemm, W., Heusinkveld, B. G., Lenzholzer, S., & van Hove, B. (2015). Street greenery and its physical and psychological impact on thermal comfort. *Landscape and Urban Planning*, *138*, 87–98. https://doi.org/10.1016/j.landurbplan.2015.02.009

Klompmaker, J. O., Janssen, N. A. H., Bloemsma, L. D., Marra, M., Lebret, E., Gehring, U., & Hoek, G. (2021). Effects of exposure to surrounding green, air pollution and traffic noise with non-accidental and cause-specific mortality in the Dutch national cohort. *Environmental Health*, *20*(1), 82.

https://doi.org/10.1186/s12940-021-00769-0

Kwon, M. Y., Lee, J. S., & Park, S. (2019). The effect of outdoor air pollutants and greenness on allergic rhinitis incidence rates: A cross-sectional study in Seoul, Korea. *International Journal of Sustainable Development & World Ecology*, *26*(3), 258–267. https://doi.org/10.1080/13504509.2019.1570982

Lai, Y., & Kontokosta, C. E. (2019). The impact of urban street tree species on air quality and respiratory illness: A spatial analysis of large-scale, high-resolution urban data. *Health & Place*, *56*, 80–87. https://doi.org/10.1016/j.healthplace.2019.01.016

Lanki, T., Siponen, T., Ojala, A., Korpela, K., Pennanen, A., Tiittanen, P., Tsunetsugu, Y., Kagawa, T., & Tyrväinen, L. (2017). Acute effects of visits to urban green environments on cardiovascular physiology in women: A field experiment. *Environmental Research*, *159*, 176–185. https://doi.org/10.1016/j.envres.2017.07.039

Lee, J.-Y., Lamichhane, D. K., Lee, M., Ye, S., Kwon, J.-H., Park, M.-S., Kim, H.-C., Leem, J.-H., Hong, Y.-C., Kim, Y., Ha, M., & Ha, E. (2018). Preventive Effect of Residential Green Space on Infantile Atopic Dermatitis Associated with Prenatal Air Pollution Exposure. *International Journal of Environmental Research and Public Health*, *15*(1), Article 1. https://doi.org/10.3390/ijerph15010102

Li, C.-C., Du, Z.-C., Fan, S.-J., Mute Browning, M. H. E., Knibbs, L. D., Bloom, M. S., Zhao, T.-Y., Jalaludin, B., Heinrich, J., Liu, X.-X., Li, J.-X., Zhang, Y.-D., Hu, L.-X., Xiang, M.-D., Chen, G.-B., Wang, Q., Han, C.-L., Li, S.-S., Guo, Y.-M., … Yang, B.-Y. (2023). Association between long-term green space exposure and mortality in China: A difference-in-differences analysis of national data in 2000, 2010 and 2019. *Science of The Total Environment*, *887*, 164023. https://doi.org/10.1016/j.scitotenv.2023.164023

Li, G., Liu, J., Lu, H., Hu, W., Hu, M., He, J., Yang, W., Zhu, Z., Zhu, J., Zhang, H., Zhao, H., & Huang, F. (2022). Multiple environmental exposures and obesity in eastern China: An individual exposure evaluation model. *Chemosphere*, *298*, 134316. https://doi.org/10.1016/j.chemosphere.2022.134316

Li, H., Browning, M. H. E. M., Dzhambov, A. M., Zhang, G., & Cao, Y. (2022). Green Space for Mental Health in the COVID-19 Era: A Pathway Analysis in Residential Green Space Users. *Land*, *11*(8), Article 8. https://doi.org/10.3390/land11081128

Li, K., Mao, Y., Li, Y., Wei, J., Shou, T., Lu, D., & Geng, W. (2024). Exploring the pathways of urban green space exposure on respiratory health: An empirical study in Nanjing, China. *Urban Forestry & Urban Greening*, *101*, 128536. https://doi.org/10.1016/j.ufug.2024.128536

Li, T., Yu, Z., Xu, L., Wu, Y., Yu, L., Yang, Z., Shen, P., Lin, H., Shui, L., Tang, M., Jin, M., Chen, K., & Wang, J. (2022). Residential greenness, air pollution, and incident ischemic heart disease: A prospective cohort study in China. *Science of The Total Environment*, *838*, 155881. https://doi.org/10.1016/j.scitotenv.2022.155881

Liang, Z., Wang, W., Yang, C., Wang, Y., Shen, J., Li, P., Ma, L., Wei, F., Chen, R., Liang, C., Li, S., & Zhang, L. (2022). Residential greenness and prevalence of chronic kidney disease: Findings from the China National Survey of Chronic Kidney Disease. *Science of The Total Environment*, *806*, 150628. https://doi.org/10.1016/j.scitotenv.2021.150628

Liao, J., Chen, X., Xu, S., Li, Y., Zhang, B., Cao, Z., Zhang, Y., Liang, S., Hu, K., & Xia, W. (2019). Effect of residential exposure to green space on maternal blood glucose levels, impaired glucose tolerance, and gestational diabetes mellitus. *Environmental Research*, *176*, 108526. https://doi.org/10.1016/j.envres.2019.108526

Liao, J., Zhang, B., Xia, W., Cao, Z., Zhang, Y., Liang, S., Hu, K., Xu, S., & Li, Y. (2019). Residential exposure to green space and early childhood neurodevelopment. *Environment International*, *128*, 70–76. https://doi.org/10.1016/j.envint.2019.03.070

Liao, L., & Du, M. (2022). Associations between Greenspaces and Individual Health: A Longitudinal Study in China. *International Journal of Environmental*

Research and Public Health, *19*(20), Article 20. https://doi.org/10.3390/ijerph192013353

Liu, C., Yu, Y., Liu, C., Tang, L., Zhao, K., Zhang, P., He, F., Wang, M., Shi, C., Lu, Z., Zhang, B., Wei, J., Xue, F., Guo, X., & Jia, X. (2023). Effect of neighbourhood greenness on the association between air pollution and risk of stroke first onset: A case-crossover study in shandong province, China. *International Journal of Hygiene and Environmental Health*, *254*, 114262. https://doi.org/10.1016/j.ijheh.2023.114262

Liu, C., Zhang, B., Liu, C., Zhang, Y., Zhao, K., Zhang, P., Tian, M., Lu, Z., Guo, X., & Jia, X. (2024). Association of ambient ozone exposure and greenness exposure with hemorrhagic stroke mortality at different times: A cohort study in Shandong Province, China. *Ecotoxicology and Environmental Safety*, *278*, 116356. https://doi.org/10.1016/j.ecoenv.2024.116356

Liu, K., Iyer, H. S., Lu, Y., Laden, F., Song, M., & Roscoe, C. (2025). Neighborhood socioeconomic disparities in cancer incidence following a hypothetical intervention to increase residential greenspace cover in the UK Biobank cohort. *Environmental Research*, *266*, 120387. https://doi.org/10.1016/j.envres.2024.120387

Liu, L., Yan, L. L., Lv, Y., Zhang, Y., Li, T., Huang, C., Kan, H., Zhang, J., Zeng, Y., Shi, X., & Ji, J. S. (2022). Air pollution, residential greenness, and metabolic dysfunction biomarkers: Analyses in the Chinese Longitudinal Healthy Longevity Survey. *BMC Public Health*, *22*(1), 885. https://doi.org/10.1186/s12889-022-13126-8

Liu, M., Yang, S., Ye, Z., Zhang, Y., He, P., Zhou, C., Zhang, Y., & Qin, X. (2023). Residential green and blue spaces with nonalcoholic fatty liver disease incidence: Mediating effect of air pollutants. *Ecotoxicology and Environmental Safety*, *264*, 115436. https://doi.org/10.1016/j.ecoenv.2023.115436

Liu, S., Qi, J., Xu, J., Yi, Y., Yin, P., & Zhou, M. (2024). Forest Mitigates Short-Term Health Risk of Air Pollution: Evidence from China. *Environmental and Resource Economics*, *87*(8), 2163–2204. https://doi.org/10.1007/s10640-024-00889-4

Liu, Y., Xiao, T., & Wu, W. (2022). Can multiple pathways link urban residential greenspace to subjective well-being among middle-aged and older Chinese adults? *Landscape and Urban Planning*, *223*, 104405. https://doi.org/10.1016/j.landurbplan.2022.104405

Ma, X., Du, M., Deng, P., Zhou, T., & Hong, B. (2024). Effects of green walls on thermal perception and cognitive performance: An indoor study. *Building and Environment*, *250*, 111180. https://doi.org/10.1016/j.buildenv.2024.111180

Malamardi, S., Lambert, K. A., Praveena, A. S., Anand, M. P., & Erbas, B. (2022). Time Trends of Greenspaces, Air Pollution, and Asthma Prevalence among Children and Adolescents in India. *International Journal of Environmental Research and Public Health*, *19*(22), Article 22. https://doi.org/10.3390/ijerph192215273

Markevych, I., Zhao, T., Fuertes, E., Marcon, A., Dadvand, P., Vienneau, D., Garcia Aymerich, J., Nowak, D., de Hoogh, K., Jarvis, D., Abramson, M. J., Accordini, S., Amaral, A. F., Bentouhami, H., Jacobsen Bertelsen, R., Boudier, A., Bono, R., Bowatte, G., Casas, L., … Heinrich, J. (2023). Residential greenspace and lung function decline over 20 years in a prospective cohort: The ECRHS study. *Environment International*, *178*, 108036. https://doi.org/10.1016/j.envint.2023.108036

Marvuglia, A., Koppelaar, R., & Rugani, B. (2020). The effect of green roofs on the reduction of mortality due to heatwaves: Results from the application of a spatial microsimulation model to four European cities. *Ecological Modelling*, *438*, 109351. https://doi.org/10.1016/j.ecolmodel.2020.109351

McDonald, R. I., Biswas, T., Chakraborty, T. C., Kroeger, T., Cook-Patton, S. C., & Fargione, J. E. (2024). Current inequality and future potential of US urban tree cover for reducing heat-related health impacts. *Npj Urban Sustainability*, *4*(1), 1–16. https://doi.org/10.1038/s42949-024-00150-3

McDonald, R. I., Kroeger, T., Zhang, P., & Hamel, P. (2020). The Value of US Urban Tree Cover for Reducing Heat-Related Health Impacts and Electricity

Consumption. *Ecosystems*, *23*(1), 137–150. https://doi.org/10.1007/s10021-019-00395-5

Mei, Y., Zhao, J., Zhou, Q., Zhao, M., Xu, J., Li, Y., Li, K., & Xu, Q. (2022). Residential greenness attenuated association of long-term air pollution exposure with elevated blood pressure: Findings from polluted areas in Northern China. *Frontiers in Public Health*, *10*. https://doi.org/10.3389/fpubh.2022.1019965

Meo, S. A., Almutairi, F. J., Abukhalaf, A. A., & Usmani, A. M. (2021). Effect of Green Space Environment on Air Pollutants PM2.5, PM10, CO, O3, and Incidence and Mortality of SARS-CoV-2 in Highly Green and Less-Green Countries. *International Journal of Environmental Research and Public Health*, *18*(24), Article 24. https://doi.org/10.3390/ijerph182413151

Mosca, F., Dotti Sani, G. M., Giachetta, A., & Perini, K. (2021). Nature-Based Solutions: Thermal Comfort Improvement and Psychological Wellbeing, a Case Study in Genoa, Italy. *Sustainability*, *13*(21), Article 21. https://doi.org/10.3390/su132111638

Motoc, I., Ginos, B. N. R., Goncalves Soares, A., Elhakeem, A., Voortman, T., Kavousi, M., Luik, A. I., Roseboom, T. J., & De Rooij, S. R. (2025). Examining associations of air pollution and green space with depressive symptoms in adults: A LongITools cross-cohort analysis. *Environmental Research*, *264*, 120321. https://doi.org/10.1016/j.envres.2024.120321

Mouly, T. A., Mishra, G. D., Hystad, P., Nieuwenhuijsen, M., & Knibbs, L. D. (2023). Residential greenspace and anxiety symptoms among Australian women living in major cities: A longitudinal analysis. *Environment International*, *179*, 108110. https://doi.org/10.1016/j.envint.2023.108110

Nguyen Thi Khanh, H., Rigau-Sabadell, M., Khomenko, S., Pereira Barboza, E., Cirach, M., Duarte-Salles, T., Nieuwenhuijsen, M., Vrijheid, M., Mueller, N., & de Bont, J. (2025). Ambient air pollution, urban green space and childhood overweight and obesity: A health impact assessment for Barcelona, Spain. *Environmental Research*, *264*, 120306. https://doi.org/10.1016/j.envres.2024.120306

Niu, J., Xiong, J., Qin, H., Wu, H., Zhang, K., Yan, J., Ye, L., & Han, G. (2023). Thermal Comfort Influences Positive Emotions but Not Negative Emotions When Visiting Green Spaces during Summer. *Forests*, *14*(8), Article 8. https://doi.org/10.3390/f14081512

Nowak, D. J., Hirabayashi, S., Doyle, M., McGovern, M., & Pasher, J. (2018). Air pollution removal by urban forests in Canada and its effect on air quality and human health. *Urban Forestry & Urban Greening*, *29*, 40–48. https://doi.org/10.1016/j.ufug.2017.10.019

O'Callaghan-Gordo, C., Espinosa, A., Valentin, A., Tonne, C., Pérez-Gómez, B., Castaño-Vinyals, G., Dierssen-Sotos, T., Moreno-Iribas, C., de Sanjose, S., Fernandez-Tardón, G., Vanaclocha-Espi, M., Chirlaque, M. D., Cirach, M., Aragonés, N., Gómez-Acebo, I., Ardanaz, E., Moreno, V., Pollan, M., Bustamante, M., … Kogevinas, M. (2020). Green spaces, excess weight and obesity in Spain. *International Journal of Hygiene and Environmental Health*, *223*(1), 45–55. https://doi.org/10.1016/j.ijheh.2019.10.007

O'Callaghan-Gordo, C., Kogevinas, M., Cirach, M., Castaño-Vinyals, G., Aragonés, N., Delfrade, J., Fernández-Villa, T., Amiano, P., Dierssen-Sotos, T., Tardon, A., Capelo, R., Peiró-Perez, R., Moreno, V., Roca-Barceló, A., Perez-Gomez, B., Vidan, J., Molina, A. J., Oribe, M., Gràcia-Lavedan, E., … Nieuwenhuijsen, M. J. (2018). Residential proximity to green spaces and breast cancer risk: The multicase-control study in Spain (MCC-Spain). *International Journal of Hygiene and Environmental Health*, *221*(8), 1097–1106. https://doi.org/10.1016/j.ijheh.2018.07.014

Odebeatu, C. C., Darssan, D., Roscoe, C., Ahmed, M., Reid, S., & Osborne, N. J. (2024). Greenspace and risk of obesity-related cancer in the UK Biobank cohort: An analysis of private residential gardens and other greenspace types. *Science of The Total Environment*, *943*, 173833. https://doi.org/10.1016/j.scitotenv.2024.173833

Odebeatu, C. C., Darssan, D., Roscoe, C., Reid, S., & Osborne, N. J. (2025). Residential greenspace indicators and metabolic syndrome in the UK Biobank

Cohort: Mediation through behavioural, environmental, social and biomarker pathways. *Environmental Research*, *283*, 122148. https://doi.org/10.1016/j.envres.2025.122148

Orioli, R., Antonucci, C., Scortichini, M., Cerza, F., Marando, F., Ancona, C., Manes, F., Davoli, M., Michelozzi, P., Forastiere, F., & Cesaroni, G. (2019). Exposure to Residential Greenness as a Predictor of Cause-Specific Mortality and Stroke Incidence in the Rome Longitudinal Study. *Environmental Health Perspectives*, *127*(2), 027002. https://doi.org/10.1289/EHP2854

Pagalan, L., Oberlander, T. F., Hanley, G. E., Rosella, L. C., Bickford, C., Weikum, W., Lanphear, N., Lanphear, B., Brauer, M., & van den Bosch, M. (2022). The association between prenatal greenspace exposure and Autism spectrum disorder, and the potentially mediating role of air pollution reduction: A population-based birth cohort study. *Environment International*, *167*, 107445. https://doi.org/10.1016/j.envint.2022.107445

Pan, R., Wang, W., Wei, N., Liu, L., Yi, W., Song, J., Cheng, J., Su, H., & Fan, Y. (2024). Does the morphology of residential greenspaces contribute to the development of a cardiovascular-healthy city? *Environmental Research*, *257*, 119280. https://doi.org/10.1016/j.envres.2024.119280

Pang, Z., Xie, B., An, Z., & Wang, L. (2024). Spatial and moderating effects of greenspace on the association between air pollution and lung cancer incidence. *Applied Geography*, *164*, 103207. https://doi.org/10.1016/j.apgeog.2024.103207

Paoin, K., Pharino, C., Vathesatogkit, P., Phosri, A., Buya, S., Ueda, K., Seposo, X. T., Ingviya, T., Saranburut, K., Thongmung, N., Yingchoncharoen, T., & Sritara, P. (2023). Associations between residential greenness and air pollution and the incident metabolic syndrome in a Thai worker cohort. *International Journal of Biometeorology*, *67*(12), 1965–1974. https://doi.org/10.1007/s00484-023-02554-9

Pascal, M., Goria, S., Wagner, V., Sabastia, M., Guillet, A., Cordeau, E., Mauclair, C., & Host, S. (2021). Greening is a promising but likely insufficient adaptation strategy to limit the health impacts of extreme heat. *Environment International*, *151*, 106441. https://doi.org/10.1016/j.envint.2021.106441

Persson, Å., Pyko, A., Lind, T., Bellander, T., Östenson, C.-G., Pershagen, G., Eriksson, C., & Lõhmus, M. (2018). Urban residential greenness and adiposity: A cohort study in Stockholm County. *Environment International*, *121*, 832–841. https://doi.org/10.1016/j.envint.2018.10.009

Qi, W., Zhang, H., Han, Y., Chen, W., Teng, Y., Chatzidiakou, L., Barratt, B., Jones, R., Kelly, F., Zhu, T., Zhang, J., Ji, J. S., Han, Y., Chen, W., Chatzidiakou, L., Yan, L., Zhang, H., Wang, Y., Cai, Y., … Zhu, T. (2024). Short-term air pollution and greenness exposures on oxidative stress in urban and peri-urban residents in Beijing: A part of AIRLESS study. *Science of The Total Environment*, *951*, 175148. https://doi.org/10.1016/j.scitotenv.2024.175148

Qiu, C., Ji, J. S., & Bell, M. L. (2021). Effect modification of greenness on temperature-mortality relationship among older adults: A case-crossover study in China. *Environmental Research*, *197*, 111112. https://doi.org/10.1016/j.envres.2021.111112

Requia, W. J., Saenger, C. C., Cicerelli, R. E., de Abreu, L. M., & Cruvinel, V. R. N. (2022). Greenness around Brazilian schools may improve students' math performance but not science performance. *Urban Forestry & Urban Greening*, *78*, 127768. https://doi.org/10.1016/j.ufug.2022.127768

Riggs, D. W., Yeager, R., Conklin, D. J., DeJarnett, N., Keith, R. J., DeFilippis, A. P., Rai, S. N., & Bhatnagar, A. (2021). Residential proximity to greenness mitigates the hemodynamic effects of ambient air pollution. *American Journal of Physiology-Heart and Circulatory Physiology*, *320*(3), H1102–H1111. https://doi.org/10.1152/ajpheart.00689.2020

Rodriguez-Loureiro, L., Verdoodt, F., Lefebvre, W., Vanpoucke, C., Casas, L., & Gadeyne, S. (2022). Long-term exposure to residential green spaces and site-specific cancer mortality in urban Belgium: A 13-year follow-up cohort study. *Environment International*, *170*, 107571. https://doi.org/10.1016/j.envint.2022.107571

Roscoe, C., Mackay, C., Gulliver, J., Hodgson, S., Cai, Y., Vineis, P., & Fecht, D. (2022). Associations of private residential gardens versus other greenspace

types with cardiovascular and respiratory disease mortality: Observational evidence from UK Biobank. *Environment International*, *167*, 107427. https://doi.org/10.1016/j.envint.2022.107427

Sadeghi, M., Chaston, T., Hanigan, I., de Dear, R., Santamouris, M., Jalaludin, B., & Morgan, G. G. (2022). The health benefits of greening strategies to cool urban environments – A heat health impact method. *Building and Environment*, *207*, 108546. https://doi.org/10.1016/j.buildenv.2021.108546

Schinasi, L. H., Bakhtsiyarava, M., Sanchez, B. N., Kephart, J. L., Ju, Y., Arunachalam, S., Gouveia, N., Teixeira Caiaffa, W., O'Neill, M. S., Dronova, I., Diez Roux, A. V., & Rodriguez, D. A. (2023). Greenness and excess deaths from heat in 323 Latin American cities: Do associations vary according to climate zone or green space configuration? *Environment International*, *180*, 108230. https://doi.org/10.1016/j.envint.2023.108230

Shen, Y.-S. (2025). Nature-based solutions to address anxiety disorders: A cross-sectional ecological study of green spatial patterns in Taiwan. *Social Science & Medicine*, *365*, 117540. https://doi.org/10.1016/j.socscimed.2024.117540

Shen, Y.-S., & Lung, S.-C. C. (2016). Can green structure reduce the mortality of cardiovascular diseases? *Science of The Total Environment*, *566–567*, 1159–1167. https://doi.org/10.1016/j.scitotenv.2016.05.159

Sheridan, S., de Guzman, E. B., Eisenman, D. P., Sailor, D. J., Parfrey, J., & Kalkstein, L. S. (2024). Increasing tree cover and high-albedo surfaces reduces heat-related ER visits in Los Angeles, CA. *International Journal of Biometeorology*. https://doi.org/10.1007/s00484-024-02688-4

Sicard, P., Pascu, I.-S., Petrea, S., Leca, S., Marco, A. D., Paoletti, E., Agathokleous, E., & Calatayud, V. (2025). Effect of tree canopy cover on air pollution-related mortality in European cities: An integrated approach. *The Lancet Planetary Health*, *9*(6), e527–e537. https://doi.org/10.1016/S2542-5196(25)00112-3

Sinha, P., Coville, R. C., Hirabayashi, S., Lim, B., Endreny, T. A., & Nowak, D. J. (2021). Modeling lives saved from extreme heat by urban tree cover☆. *Ecological Modelling*, *449*, 109553. https://doi.org/10.1016/j.ecolmodel.2021.109553

Son, J.-Y., Lane, K. J., Lee, J.-T., & Bell, M. L. (2016). Urban vegetation and heat-related mortality in Seoul, Korea. *Environmental Research*, *151*, 728–733. https://doi.org/10.1016/j.envres.2016.09.001

Song, J., Gasparrini, A., Wei, D., Lu, Y., Hu, K., Fischer, T. B., & Nieuwenhuijsen, M. (2024). Do greenspaces really reduce heat health impacts? Evidence for different vegetation types and distance-based greenspace exposure. *Environment International*, *191*, 108950. https://doi.org/10.1016/j.envint.2024.108950

Song, S., Xiao, Y., Tu, R., & Yin, S. (2024). Effects of thermal perception on restorative benefits by green space exposure: A pilot study in hot-humid China. *Urban Climate*, *53*, 101767. https://doi.org/10.1016/j.uclim.2023.101767

Sun, H., Pan, C., Yan, M., Wang, Z., He, J., Zhang, H., Yang, Z., Wang, Z., Wang, Y., Liu, H., Yang, X., Hou, F., Wei, J., Yu, P., Chen, X., & Tang, N.-J. (2025). Effects of PM2.5 components on hypertension and diabetes: Assessing the mitigating influence of green spaces. *Science of The Total Environment*, *959*, 178219. https://doi.org/10.1016/j.scitotenv.2024.178219

Sun, S., Sarkar, C., Kumari, S., James, P., Cao, W., Lee, R. S., Tian, L., & Webster, C. (2020). Air pollution associated respiratory mortality risk alleviated by residential greenness in the Chinese Elderly Health Service Cohort. *Environmental Research*, *183*, 109139. https://doi.org/10.1016/j.envres.2020.109139

Sun, X., Luo, X., Cao, G., Zhao, C., Xiao, J., Liu, X., Dong, M., Wang, J., Zeng, W., Guo, L., Wan, D., Ma, W., & Liu, T. (2020). Associations of ambient temperature exposure during pregnancy with the risk of miscarriage and the modification effects of greenness in Guangdong, China. *Science of The*

Total Environment, *702*, 134988. https://doi.org/10.1016/j.scitotenv.2019.134988

Sun, Y., Sheridan, P., Laurent, O., Li, J., Sacks, D. A., Fischer, H., Qiu, Y., Jiang, Y., Yim, I. S., Jiang, L.-H., Molitor, J., Chen, J.-C., Benmarhnia, T., Lawrence, J. M., & Wu, J. (2020). Associations between green space and preterm birth: Windows of susceptibility and interaction with air pollution. *Environment International*, *142*, 105804. https://doi.org/10.1016/j.envint.2020.105804

Sung, W.-P., Liao, M.-C., Peng, H.-L., Huang, C.-T., Chuang, Y.-J., & Wang, J.-J. (2025). The Role of Urban Food Forests in Promoting Environmental Sustainability and Public Health: A Focus on Temperature Regulation and Mental Health. *Sustainability*, *17*(7), Article 7. https://doi.org/10.3390/su17072875

Tang, P., Liu, T., Zheng, X., & Zheng, J. (2025). Spatiotemporal Dynamics of PM2.5-Related Premature Deaths and the Role of Greening Improvement in Sustainable Urban Health Governance. *Atmosphere*, *16*(2), Article 2. https://doi.org/10.3390/atmos16020232

Taylor, J., Simpson, C., Brousse, O., Viitanen, A.-K., & Heaviside, C. (2024). The potential of urban trees to reduce heat-related mortality in London. *Environmental Research Letters*, *19*(5), 054004. https://doi.org/10.1088/1748-9326/ad3a7e

Terre-Torras, I., Recalde, M., Díaz, Y., de Bont, J., Bennett, M., Aragón, M., Cirach, M., O'Callaghan-Gordo, C., Nieuwenhuijsen, M. J., & Duarte-Salles, T. (2022). Air pollution and green spaces in relation to breast cancer risk among pre and postmenopausal women: A mega cohort from Catalonia. *Environmental Research*, *214*, 113838. https://doi.org/10.1016/j.envres.2022.113838

Thiering, E., Markevych, I., Brüske, I., Fuertes, E., Kratzsch, J., Sugiri, D., Hoffmann, B., von Berg, A., Bauer, C.-P., Koletzko, S., Berdel, D., & Heinrich, J. (2016). Associations of Residential Long-Term Air Pollution Exposures and Satellite-Derived Greenness with Insulin Resistance in German Adolescents. *Environmental Health Perspectives*, *124*(8), 1291–1298. https://doi.org/10.1289/ehp.1509967

Torres Toda, M., Miri, M., Heydari, H., Lari Najafi, M., Gómez-Roig, M. D., Llurba, E., Foraster, M., & Dadvand, P. (2022). A study on exposure to greenspace during pregnancy and lipid profile in cord blood samples. *Environmental Research*, *214*, 113732. https://doi.org/10.1016/j.envres.2022.113732

Triebner, K., Markevych, I., Bertelsen, R. J., Sved Skottvoll, B., Hustad, S., Forsberg, B., Franklin, K. A., Holm, M., Lindberg, E., Heinrich, J., Gómez Real, F., & Dadvand, P. (2022). Lifelong exposure to residential greenspace and the premenstrual syndrome: A population-based study of Northern European women. *Environment International*, *158*, 106975. https://doi.org/10.1016/j.envint.2021.106975

Vienneau, D., de Hoogh, K., Faeh, D., Kaufmann, M., Wunderli, J. M., & Röösli, M. (2017). More than clean air and tranquillity: Residential green is independently associated with decreasing mortality. *Environment International*, *108*, 176–184. https://doi.org/10.1016/j.envint.2017.08.012

Vranken, A., Bijnens, E., Horemans, C., Leclercq, A., Kestens, W., Karakaya, G., Vandenthoren, L., Trimpeneers, E., Vanpoucke, C., Fierens, F., Nawrot, T., Cox, B., & Bruyneel, L. (2023). Association of air pollution and green space with all-cause general practitioner and emergency room visits: A cross-sectional study of young people and adults living in Belgium. *Environmental Research*, *236*, 116713. https://doi.org/10.1016/j.envres.2023.116713

Wan, S., Rojas-Rueda, D., Pretty, J., Roscoe, C., James, P., & Ji, J. S. (2022). Greenspace and mortality in the U.K. Biobank: Longitudinal cohort analysis of socio-economic, environmental, and biomarker pathways. *SSM - Population Health*, *19*, 101194. https://doi.org/10.1016/j.ssmph.2022.101194

Wang, D., Zhang, Y., Dong, X., Hu, Y., Ma, W., Li, N., Chang, J., & Wang, Y. (2025). Sensitive months for green spaces' impact on macrosomia and interaction with air pollutants: A birth cohort study. *Environmental Pollution*, *368*, 125743. https://doi.org/10.1016/j.envpol.2025.125743

Wang, G., Yang, F. F., Lin, G., Wang, Z., & Zhang, X. (2023). Modification of low temperature-related hospital admissions for cardiovascular diseases by multiple green space indicators at multiple spatial scales: Evidence from Guangzhou, China. *International Journal of Hygiene and Environmental*

Health, *251*, 114193. https://doi.org/10.1016/j.ijheh.2023.114193

Wang, R., Dong, P., Dong, G., Xiao, X., Huang, J., Yang, L., Yu, Y., & Dong, G.-H. (2022). Exploring the impacts of street-level greenspace on stroke and cardiovascular diseases in Chinese adults. *Ecotoxicology and Environmental Safety*, *243*, 113974. https://doi.org/10.1016/j.ecoenv.2022.113974

Wang, R., Feng, Z., Pearce, J., Liu, Y., & Dong, G. (2021). Are greenspace quantity and quality associated with mental health through different mechanisms in Guangzhou, China: A comparison study using street view data. *Environmental Pollution*, *290*, 117976. https://doi.org/10.1016/j.envpol.2021.117976

Wu, L., & Chen, C. (2023). Does pattern matter? Exploring the pathways and effects of urban green space on promoting life satisfaction through reducing air pollution. *Urban Forestry & Urban Greening*, *82*, 127890. https://doi.org/10.1016/j.ufug.2023.127890

Wu, W., Wu, G., Wei, J., Lawrence, W. R., Deng, X., Zhang, Y., Chen, S., Wang, Y., Lin, X., Chen, D., Ruan, X., Lin, Q., Li, Z., Lin, Z., Hao, C., Du, Z., Zhang, W., & Hao, Y. (2024). Potential causal links and mediation pathway between urban greenness and lung cancer mortality: Result from a large cohort (2009 to 2020). *Sustainable Cities and Society*, *101*, 105079. https://doi.org/10.1016/j.scs.2023.105079

Xiao, X., Wang, R., Knibbs, L. D., Jalaludin, B., Heinrich, J., Markevych, I., Gao, M., Xu, S.-L., Wu, Q.-Z., Zeng, X.-W., Chen, G.-B., Hu, L.-W., Yang, B.-Y., Yu, Y., & Dong, G.-H. (2021). Street view greenness is associated with lower risk of obesity in adults: Findings from the 33 Chinese community health study. *Environmental Research*, *200*, 111434. https://doi.org/10.1016/j.envres.2021.111434

Xiao, X., Yang, B.-Y., Hu, L.-W., Markevych, I., Bloom, M. S., Dharmage, S. C., Jalaludin, B., Knibbs, L. D., Heinrich, J., Morawska, L., Lin, S., Roponen, M., Guo, Y., Lam Yim, S. H., Leskinen, A., Komppula, M., Jalava, P., Yu, H.-Y., Zeeshan, M., … Dong, G.-H. (2020). Greenness around schools associated with lower risk of hypertension among children: Findings from the Seven Northeastern Cities Study in China. *Environmental Pollution*, *256*, 113422. https://doi.org/10.1016/j.envpol.2019.113422

Xiao, Y., Liu, C., Lei, R., Wang, Z., Wang, X., Tian, H., Xue, B., Zhou, E., Zhang, K., Hu, J., & Luo, B. (2023). Associations of PM2.5 composition and green space with metabolic syndrome in a Chinese essential hypertensive population. *Chemosphere*, *343*, 140243. https://doi.org/10.1016/j.chemosphere.2023.140243

Xu, S., Marcon, A., Bertelsen, R. J., Benediktsdottir, B., Brandt, J., Frohn, L. M., Geels, C., Gislason, T., Heinrich, J., Holm, M., Janson, C., Markevych, I., Modig, L., Orru, H., Schlünssen, V., Sigsgaard, T., & Johannessen, A. (2025). Long-term exposure to air pollution and greenness in association with respiratory emergency room visits and hospitalizations: The Life-GAP project. *Environmental Research*, *270*, 120938. https://doi.org/10.1016/j.envres.2025.120938

Xue, E., Zhao, J., Ye, J., Wu, J., Chen, D., Shao, J., Li, X., & Ye, Z. (2025). Green sanctuaries: Residential green and garden space and the natural environment mitigate mental disorders risk of diabetic patients. *BMC Medicine*, *23*(1), 31. https://doi.org/10.1186/s12916-025-03864-y

Yang, C., Wang, J., Yang, H., Liao, J., Wang, X., Jiao, K., Ma, X., Liao, J., Liu, X., & Ma, L. (2022). Association of NO2 with daily hospital admissions for mental disorders: Investigation of the modification effects of green spaces and long-term NO2 exposure. *Journal of Psychiatric Research*, *156*, 698–704. https://doi.org/10.1016/j.jpsychires.2022.11.014

Yang, L., Yang, Z., Zhao, Z., Norbäck, D., Cai, Y. S., & Zhang, X. (2023). Exposure to greenness, air pollution and respiratory health among pre-school children in northern China. *Atmospheric Environment*, *298*, 119608. https://doi.org/10.1016/j.atmosenv.2023.119608

Yang, T., Gu, T., Xu, Z., He, T., Li, G., & Huang, J. (2023). Associations of residential green space with incident type 2 diabetes and the role of air pollution: A prospective analysis in UK Biobank. *Science of The Total Environment*, *866*, 161396. https://doi.org/10.1016/j.scitotenv.2023.161396

Yang, X., Duan, C., Chen, B., & Wang, H. (2025). The socio-economic value of urban green spaces in mitigating waterlogging and enhancing well-being. *Resources, Conservation and Recycling*, *212*, 108010. https://doi.org/10.1016/j.resconrec.2024.108010

Ye, Z., Liu, M., He, P., Wu, Q., Yang, S., Zhang, Y., Zhou, C., Zhang, Y., Gan, X., Sun, J., & Qin, X. (2023). Various ambient air pollutants, residential green spaces, fibrosis 4 scores, genetic susceptibility, and risk of severe liver disease. *Ecotoxicology and Environmental Safety*, *263*, 115246. https://doi.org/10.1016/j.ecoenv.2023.115246

Yi, L., Hart, J. E., Roscoe, C., Mehta, U. V., Pescador Jimenez, M., Lin, P.-I. D., Suel, E., Hystad, P., Hankey, S., Zhang, W., Okereke, O. I., Laden, F., & James, P. (2025). Greenspace and depression incidence in the US-based nationwide Nurses' Health Study II: A deep learning analysis of street-view imagery. *Environment International*, *198*, 109429. https://doi.org/10.1016/j.envint.2025.109429

Yu, B., Tang, W., Fan, Y., Ma, C., Ye, T., Cai, C., Xie, Y., Shi, Y., Baima, K., Yang, T., Wang, Y., Jia, P., & Yang, S. (2024). Associations between residential greenness and obesity phenotypes among adults in Southwest China. *Health & Place*, *87*, 103236. https://doi.org/10.1016/j.healthplace.2024.103236

Yu, H., Zhou, Y., Wang, R., Qian, Z., Knibbs, L. D., Jalaludin, B., Schootman, M., McMillin, S. E., Howard, S. W., Lin, L.-Z., Zhou, P., Hu, L.-W., Liu, R.-Q., Yang, B.-Y., Chen, G., Zeng, X.-W., Feng, W., Xiang, M., & Dong, G.-H. (2021). Associations between trees and grass presence with childhood asthma prevalence using deep learning image segmentation and a novel green view index. *Environmental Pollution*, *286*, 117582. https://doi.org/10.1016/j.envpol.2021.117582

Yu, K., Zhang, Q., Meng, X., Zhang, L., Kan, H., & Chen, R. (2023). Association of residential greenness with incident chronic obstructive pulmonary disease: A prospective cohort study in the UK Biobank. *Environment International*, *171*, 107654. https://doi.org/10.1016/j.envint.2022.107654

Yu, W., Liu, Z., La, Y., Feng, C., Yu, B., Wang, Q., Liu, M., Li, Z., Feng, Y., Ciren, L., Zeng, Q., Zhou, J., Zhao, X., Jia, P., & Yang, S. (2023). Associations between residential greenness and the predicted 10-year risk for atherosclerosis cardiovascular disease among Chinese adults. *Science of The Total Environment*, *868*, 161643. https://doi.org/10.1016/j.scitotenv.2023.161643

Yu, Y., Lin, H., Liu, Q., Ma, Y., Zhao, L., Li, W., Zhou, Y., Byun, H.-M., Li, P., Li, C., Sun, C., Chen, X., Liu, Z., Dong, W., Chen, L., Deng, F., Wu, S., Hou, S., & Guo, L. (2024). Association of residential greenness, air pollution with adverse birth outcomes: Results from 61,762 mother-neonatal pairs in project ELEFANT (2011–2021). *Science of The Total Environment*, *912*, 169549. https://doi.org/10.1016/j.scitotenv.2023.169549

Yu, Z., Feng, Y., Chen, Y., Zhang, X., Zhao, X., Chang, H., Zhang, J., Gao, Z., Zhang, H., & Huang, C. (2023). Green space, air pollution and gestational diabetes mellitus: A retrospective cohort study in central China. *Ecotoxicology and Environmental Safety*, *249*, 114457. https://doi.org/10.1016/j.ecoenv.2022.114457

Yu, Z., Wei, F., Zhang, X., Wu, M., Lin, H., Shui, L., Jin, M., Wang, J., Tang, M., & Chen, K. (2021). Air pollution, surrounding green, road proximity and Parkinson's disease: A prospective cohort study. *Environmental Research*, *197*, 111170. https://doi.org/10.1016/j.envres.2021.111170

Yuan, Y., Zhou, P., Peng, M., Zhu, L., Li, Y., Wang, K., Wang, Y., Tang, Z., Wang, Y., Huang, Y., Zhang, J., & Zhang, Y. (2023). Residential greenness mitigates mortality risk from short-term airborne particulate exposure: An individual-level case-crossover study. *Ecotoxicology and Environmental Safety*, *264*, 115451. https://doi.org/10.1016/j.ecoenv.2023.115451

Yuchi, W., Brauer, M., Czekajlo, A., Davies, H. W., Davis, Z., Guhn, M., Jarvis, I., Jerrett, M., Nesbitt, L., Oberlander, T. F., Sbihi, H., Su, J., & van den Bosch, M. (2022). Neighborhood environmental exposures and incidence of attention deficit/hyperactivity disorder: A population-based cohort study. *Environment International*, *161*, 107120. https://doi.org/10.1016/j.envint.2022.107120

Zhang, J., Yang, Z., Sun, Y., Xu, Z., Hui, T., & Guo, P. (2023). Experiencing urban forests for mitigation of negative emotions of people exposed to seasonal PM2.5 in Northeast China. *Journal of Forestry Research*, *34*(5), 1245–1261. https://doi.org/10.1007/s11676-022-01595-x

Zhang, T., Jiang, W., Huang, Y., Wang, C., Cao, J., Qiu, W., Huang, R., Zhang, J., Ye, W., & Zhang, Q. (2025). The influence of socioeconomic status on the association between residential greenness and gestational diabetes mellitus in an urban setting: A multicenter study. *BMC Public Health*, *25*(1), 1708. https://doi.org/10.1186/s12889-025-22913-y

Zhang, Y., Wang, D., Xu, R., Lu, P., Dong, X., Ma, W., Hu, Y., Tian, Y., Liu, Y., Zhou, S., & Guo, Y. (2025). Moderating effect of green space on relationship between atmospheric particulate matter and cardiovascular and cerebrovascular disease mortality in Ningxia, China. *Environmental Research*, *270*, 120931. https://doi.org/10.1016/j.envres.2025.120931

Zhao, C., Wang, W., Wen, H., Huang, Z., Wang, X., Jiao, K., Chen, Q., Feng, H., Wang, Y., Liao, J., & Ma, L. (2023). Effects of green spaces on alleviating mortality attributable to PM2.5 in China. *Environmental Science and Pollution Research*, *30*(6), 14402–14412. https://doi.org/10.1007/s11356-022-23097-3

Zhao, X., Wang, J., Wu, Z., Li, H., Li, Z., Liu, Y., Li, X., Guo, X., & Tao, L. (2025). Association of residential green space with risk of sarcopenia and the role of air pollution: Evidence from UK Biobank. *Environmental Pollution*, *370*, 125857. https://doi.org/10.1016/j.envpol.2025.125857

Zhou, F., Xie, B., Liu, K., & Chen, B. (2025). Exploring the interactive effects of greenspace morphology and air pollutant on tuberculosis treatment outcomes: A comparative analysis between urban and rural areas in China. *Urban Forestry & Urban Greening*, *104*, 128676. https://doi.org/10.1016/j.ufug.2025.128676

Zhou, S., Guo, Y., Bao, Z., Lin, L., Liu, H., Chen, G., Li, Q., Bao, H., Ji, Y., Luo, S., Liu, Z., Wang, H., Han, N., & Wang, H.-J. (2022). Individual and joint effects of prenatal green spaces, PM2.5 and PM1 exposure on BMI Z-score of children aged two years: A birth cohort study. *Environmental Research*, *205*, 112548. https://doi.org/10.1016/j.envres.2021.112548

Zhou, W., Wang, Q., Kadier, A., Wang, W., Zhou, F., Li, R., & Ling, L. (2023). The role of residential greenness levels, green land cover types and diversity in overweight/obesity among older adults: A cohort study. *Environmental Research*, *217*, 114854. https://doi.org/10.1016/j.envres.2022.114854

Zhou, W., Wang, Q., Li, R., Kadier, A., Wang, W., Zhou, F., & Ling, L. (2023). Combined effects of heatwaves and air pollution, green space and blue space on the incidence of hypertension: A national cohort study. *Science of The Total Environment*, *867*, 161560. https://doi.org/10.1016/j.scitotenv.2023.161560

Zhu, Z., Yang, Z., Yu, L., Xu, L., Wu, Y., Zhang, X., Shen, P., Lin, H., Shui, L., Tang, M., Jin, M., Wang, J., & Chen, K. (2023). Residential greenness, air pollution and incident neurodegenerative disease: A cohort study in China. *Science of The Total Environment*, *878*, 163173. https://doi.org/10.1016/j.scitotenv.2023.163173
